# Population comparative genomics discovers gene gain and loss during grapevine domestication

**DOI:** 10.1093/plphys/kiae039

**Published:** 2024-01-29

**Authors:** Qiming Long, Shuo Cao, Guizhou Huang, Xu Wang, Zhongjie Liu, Wenwen Liu, Yiwen Wang, Hua Xiao, Yanling Peng, Yongfeng Zhou

**Affiliations:** National Key Laboratory of Tropical Crop Breeding, Shenzhen Branch, Guangdong Laboratory of Lingnan Modern Agriculture, Key Laboratory of Synthetic Biology, Ministry of Agriculture and Rural Affairs, Agricultural Genomics Institute at Shenzhen, Chinese Academy of Agricultural Sciences, Shenzhen, 518124, China; National Key Laboratory of Tropical Crop Breeding, Shenzhen Branch, Guangdong Laboratory of Lingnan Modern Agriculture, Key Laboratory of Synthetic Biology, Ministry of Agriculture and Rural Affairs, Agricultural Genomics Institute at Shenzhen, Chinese Academy of Agricultural Sciences, Shenzhen, 518124, China; Key Laboratory of Horticultural Plant Biology Ministry of Education, Huazhong Agricultural University, Wuhan, 430070, China; National Key Laboratory of Tropical Crop Breeding, Shenzhen Branch, Guangdong Laboratory of Lingnan Modern Agriculture, Key Laboratory of Synthetic Biology, Ministry of Agriculture and Rural Affairs, Agricultural Genomics Institute at Shenzhen, Chinese Academy of Agricultural Sciences, Shenzhen, 518124, China; National Key Laboratory of Tropical Crop Breeding, Shenzhen Branch, Guangdong Laboratory of Lingnan Modern Agriculture, Key Laboratory of Synthetic Biology, Ministry of Agriculture and Rural Affairs, Agricultural Genomics Institute at Shenzhen, Chinese Academy of Agricultural Sciences, Shenzhen, 518124, China; School of Agriculture and Food Science, University College Dublin, Belfield, Dublin, D04 C1P1, Ireland; National Key Laboratory of Tropical Crop Breeding, Shenzhen Branch, Guangdong Laboratory of Lingnan Modern Agriculture, Key Laboratory of Synthetic Biology, Ministry of Agriculture and Rural Affairs, Agricultural Genomics Institute at Shenzhen, Chinese Academy of Agricultural Sciences, Shenzhen, 518124, China; National Key Laboratory of Tropical Crop Breeding, Shenzhen Branch, Guangdong Laboratory of Lingnan Modern Agriculture, Key Laboratory of Synthetic Biology, Ministry of Agriculture and Rural Affairs, Agricultural Genomics Institute at Shenzhen, Chinese Academy of Agricultural Sciences, Shenzhen, 518124, China; National Key Laboratory of Tropical Crop Breeding, Shenzhen Branch, Guangdong Laboratory of Lingnan Modern Agriculture, Key Laboratory of Synthetic Biology, Ministry of Agriculture and Rural Affairs, Agricultural Genomics Institute at Shenzhen, Chinese Academy of Agricultural Sciences, Shenzhen, 518124, China; National Key Laboratory of Tropical Crop Breeding, Shenzhen Branch, Guangdong Laboratory of Lingnan Modern Agriculture, Key Laboratory of Synthetic Biology, Ministry of Agriculture and Rural Affairs, Agricultural Genomics Institute at Shenzhen, Chinese Academy of Agricultural Sciences, Shenzhen, 518124, China; National Key Laboratory of Tropical Crop Breeding, Shenzhen Branch, Guangdong Laboratory of Lingnan Modern Agriculture, Key Laboratory of Synthetic Biology, Ministry of Agriculture and Rural Affairs, Agricultural Genomics Institute at Shenzhen, Chinese Academy of Agricultural Sciences, Shenzhen, 518124, China; National Key Laboratory of Tropical Crop Breeding, Shenzhen Branch, Guangdong Laboratory of Lingnan Modern Agriculture, Key Laboratory of Synthetic Biology, Ministry of Agriculture and Rural Affairs, Agricultural Genomics Institute at Shenzhen, Chinese Academy of Agricultural Sciences, Shenzhen, 518124, China; National Key Laboratory of Tropical Crop Breeding, Tropical Crops Genetic Resources Institute, Chinese Academy of Tropical Agricultural Sciences, Haikou, 571101, China

## Abstract

Plant domestication are evolutionary experiments conducted by early farmers since thousands years ago, during which the crop wild progenitors are artificially selected for desired agronomic traits along with dramatic genomic variation in the course of moderate to severe bottlenecks. However, previous investigations are mainly focused on small-effect variants, while changes in gene contents are rarely investigated due to the lack of population-level assemblies for both the crop and its wild relatives. Here, we applied comparative genomic analyses to discover gene gain and loss during grapevine domestication using long-read assemblies of representative population samples for both domesticated grapevines (*V. vinifera* ssp. *vinifera*) and their wild progenitors (*V. vinifera* ssp. *sylvestris*). Only ∼7% of gene families were shared by 16 *Vitis* genomes while ∼8% of gene families were specific to each accession, suggesting dramatic variations of gene contents in grapevine genomes. Compared to wild progenitors, the domesticated accessions exhibited an increased presence of genes associated with asexual reproduction, while the wild progenitors showcased a higher abundance of genes related to pollination, revealing the transition from sexual reproduction to clonal propagation during domestication processes. Moreover, the domesticated accessions harbored fewer disease-resistance genes than wild progenitors. The SVs occurred frequently in aroma and disease-resistance related genes between domesticated grapevines and wild progenitors, indicating the rapid diversification of these genes during domestication. Our study provides insights and resources for biological studies and breeding programs in grapevine.

## Teaser

Comparative genomic analyses of wild and cultivated grapevine at the population level reveal dramatic variations in gene content during grapevine domestication.

## Introduction

Plant domestication has been conducted by early farmers since thousands of years ago, during which the crop wild progenitors were artificially selected for desired agronomic traits along with moderate to severe bottleneck (Doebley et al. 2006; Gaut et al. 2018; Huang et al. 2022). The artificial selection aims to select the human-favored traits, leading to the increase of allele frequency, and sometimes even fixation of the beneficial alleles (Doebley et al. 2006; Gaut et al. 2018). However, at the same time, domestication brings in deleterious variants in the crop genomes due to genetic bottleneck, hitchhiking, and the relaxation of selection (Gaut et al. 2018). Previous investigations have revealed the accumulation of deleterious SNP variants (Liu et al. 2017; Zhou et al. 2017; Gaut et al. 2018; Zhang et al. 2021a) and large structural variants (SV, >50 bp) (Zhou et al. 2019a; Kou et al. 2020) in crop genomes. Such SVs that occurred during domestication could lead to a change in gene content (Zhou et al. 2019a; Tang et al. 2022), leading to the gain and loss of genes in the domesticated population compared with its wild progenitors. The gained and lost genes are often associated with important agronomic traits and biotic/abiotic resistance, which can be used as targets for future crop breeding. For example, flavor genes were lost during the domestication of tomato (*Solanum lycopersicum*) (Zhu et al. 2018).

The swift development of sequencing technologies and assembly algorithms in past decades has enabled the comparative analyses of high-quality genome assemblies based on long-reads sequencing at population level and thus the detection of big SVs in population samples. For example, the gene presence/absence has been investigated by long-read based assemblies in domesticated rice (*Oryza sativa*) (Kou et al. 2020; Shang et al. 2022), maize (*Zea mays*) (Hufford et al. 2021), sorghum (*Sorghum bicolor*) (Tao et al. 2021), soybean (*Glycine max*) (Liu et al. 2020), tomato (Zhou et al. 2022), and potato (*Solanum tuberosum*) (Tang et al. 2022) based on the comparative genomic analyses, which can efficiently identify the gene gain and loss caused by big SVs, and thus facilitate crop breeding.

Grapevine (*Vitis vinifera*) is one of the most economically important fruit crops, which has a vital impact on human culture, history and humanity. Previous studies on the origin of grapevine suggested that cultivated grapevines were domesticated from the Eurasian grapevine (*V. vinifera* ssp. *sylvestris*), the wild ancestor of grapevine, more than 10,000 years ago (Zhou et al. 2017). As a result of domestication, cultivated grapevines harbor the traits that are beneficial to mankind, including increased sugar content, enlarged berry and bunch size, pleasant aroma, and reduced seeds (This et al. 2006; Zhou et al. 2017). Plenty of population genetic analyses have been conducted and found the changes in genome-wide genetic diversity and artificial selection at specific genomic regions resulting from domestication (Zhou et al. 2017; Liang et al. 2019; Zhou et al. 2019a). However, the genes gain and loss caused by large SVs during grapevine domestication still remain unknown, due to the lack of population long-read sequencing or assemblies.

Here, we first unified 17 chromosome-level grapevine genome assemblies using long-read sequencing, including one outgroup, muscadine (*Vitis rotundifolia*, Vmu) and 16 accessions representing three *Vitis* populations (Supplementary Table S1). The cultivar population contained nine representative accessions (*V. vinifera* ssp. *vinifera*, hereafter vinifera), including two table grapes, Black Corinth Seedless, Black Corinth Seeded, and seven vine grapes, Cabernet Sauvignon, Carménère, Chardonnay, Merlot, Riesling, Zinfandel, and Pinot Noir. The wild progenitor population included five accessions (*V. vinifera* ssp*. sylvestris*, hereafter sylvestris). The wild relative population has two samples of grapevine wild relatives, riverbank grape (*V. riparia*, Vri) and Arizona grape (*V. arizonica*, Var). Then, we conducted comparative genomic analyses for the wild progenitor population, the cultivar population, and all our samples, respectively. Finally, we explored their gain/loss and gene contents changes from the wild progenitor population to the cultivar population along with their biological functions. Altogether, our results provide highly valuable resources for functional genomic studies and breeding in grapevine.

## Results

### Chromosome-level genome assembly and annotation

To investigate the gained and lost genes caused by SVs during grapevine domestication, we first collected and constructed chromosome-level genome assemblies for 17 grapevine accessions. Among these accessions, Vmu was regarded as outgroup of the remaining 16 accessions as previous studies did (Zhou et al. 2017). The remaining 16 accession, which we referred to *Vitis* population later, represented one wild progenitor population, one cultivar population and one wild relative population. Across these 17 genomes, an average of 11.15 Mb scaffold N50 length was detected (Supplementary Table S2). Benchmarking universal single-copy orthologs (BUSCO) evaluation showed that a mean of 95.08% of the 1,614 single-copy Embryophyta genes were assembled in these genomes’ primary (or haplotype1) assemblies (Supplementary Fig. S1), indicating the high credibility of these genomes. Genomic collinearity analysis revealed an overall high collinearity between our genomes (Supplementary Fig. S2). Consistent with previous findings (Cochetel et al. 2021), our results showed that chromosome 20 of Vmu was collinear with chromosome 7 of *Vitis*, so we linked chromosome 7 and chromosome 20 of Vmu for downstream analysis.

In order to eliminate the artificial effects in genome annotations caused by different pipelines, we used the uniform pipeline for the reannotation of all genomes (see Methods). As a result, an average of 32,824 protein-coding genes were predicted across the genomes. Since transposable elements (TEs) constitute a large portion of the genome in many organisms (Wells and Feschotte 2020), we further explored whether TEs contributed to the genome size variations during grapevine domestication. Meanwhile, we also used a general pipeline to reannotate TEs and found that TEs occupied 46.55% of the genome on average, with *Gypsy* and *Copia* superfamilies being the most numerous TE classes (Supplementary Fig. S3, Supplementary Table S3), which were consistent with previous study (Zhou et al. 2019a). Then we compared the genome sizes among the wild progenitors, cultivars, and wild relative populations (Supplementary Fig. S4A). We found that genome size variations can be primarily explained by the differences in TEs content (Supplementary Fig. S4B).

### Comparative genomic analyses of cultivated and wild grapes

**Figure 1.**
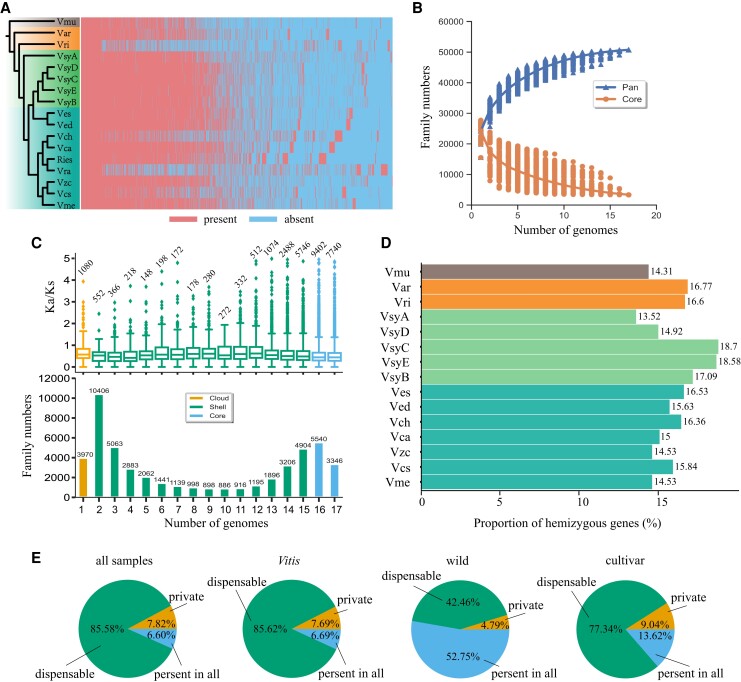
Grapevine pan-genome analyses. We clustered re-annotated genes from 17 genomes into gene families to perform comparative genomic analyses of grapevine. **A)** Phylogenetic relationships inferred from SNPs among 17 genomes along with presence/absence information of pan gene families in 17 grape genomes. **B)** Count of gene families in the pan-genome and core genome in randomly chose genomes. **C)** Ka/Ks for gene families of different frequencies and the number of gene families presented versus the number of genomes. The histogram shows the number of gene families in the 17 genomes with different frequencies, and the box plot shows the Ka/Ks values. Center line, median; box limits, upper and lower quartiles; whiskers, 1.5× interquartile range; points, outliers; numbers at the top, sample number. **D)** Proportion of hemizygous genes for each accession. (Data for Ries and Vra are missing because we failed to collect their PacBio reads.) **E)** Proportion of gene families that are present in all accessions, dispensable (present in at least two accessions but not in all) or private (only present in a single accession) in pan-genome of all samples, *Vitis* population, wild population, and cultivar population, respectively.

Firstly, we constructed the gene-based pan-genome of 17 samples by clustering the predicted gene models from our annotations using the Markov clustering algorithm (Fig. 1A). Ortholog investigation classified all genes from 17 genomes into 50,750 families. The number of pan gene families increased rapidly to a platform with the increasing genome numbers, while the number of core gene families decreased with a similar tendency (Fig. 1B), indicating the representativeness of our pan-genome. The core gene families were defined as gene families presented across more than 15 accessions, while shell gene families were defined as 2 to 15 accessions and cloud as only one (Fig. 1, C and E). The higher Ka/Ks value in cloud genes than core genes may suggest the functional conservation of the latter (Fig. 1C). Moreover, Gene Ontology (GO) annotation suggested the 8,886 core gene families (17.51% of 50,750 families) were mainly enriched in basic molecular functions such as ATP binding, RNA-directed DNA polymerase activity and protein serine kinase activity (Supplementary Fig. S5A). Whereas, the 3,970 cloud gene families (7.8%) were mainly enriched in biological processes, such as proteolysis, DNA integration, and DNA recombination (Supplementary Fig. S5B). The comparison between the outgroup (Vmu) and the combined *Vitis* population (*n* = 16) found that 751 gene families (1.48%) have remarkably more genes in the outgroup, while 479 gene families (0.94%) have remarkably more genes in the *Vitis* (Mann–Whitney–Wilcoxon Test, *P*-value < 0.05).

To explore the changes in gene families during grapevine domestication, we constructed pan-genomes of the wild progenitor population (*n* = 5, Supplementary Fig. S6, A and B), cultivar population (*n* = 9, Supplementary Fig. S6, C and D), and *Vitis* population (*n* = 16, Supplementary Fig. S6, E and F), respectively. In the wild progenitor population, a total of 20,303 gene families (52.75% of 38,485 families) containing 155,294 genes were shared between all accessions, while 1,843 gene families (4.79%) containing 5,016 genes were specific to each accession (Fig. 1E). In the cultivar population, a total of 6,289 gene families (13.62%) consisting of 117,335 genes were presented in all accessions (Fig. 1E), the functions of which were mainly related to cell wall-related process and photosynthesis-related process (Supplementary Fig. S7), while 4,173 gene families (9.04%) consisting of 12,097 genes were unique to each accession (Fig. 1E), and their functions were mainly involved in cellular aromatic compound metabolic process (Supplementary Fig. S7). In the *Vitis* population, 3,435 gene families (6.69%) consisting of 151,366 (22.21%) genes occurred in all accessions, while 3,944 gene families (7.69%) consisting of 10,631 (1.52%) genes were private (Fig. 1E).

Furthermore, we compared the gene families contained in the two haplotypes in three diploid genomes, respectively (Supplementary Fig. S8). On average, 3,725 gene families (20.29%) were different between two haplotypes of diploid genomes. We also detected the proportion of hemizygous genes which were defined as the genes that were surrounded by a heterozygous insertion/deletion variation (Fig. 1D), and found a comparable results to the gene family analyses and previous analyses in Chardonnay (Zhou et al. 2019a). The proportion of hemizygous genes in all accessions ranged from 13.52% to 18.70%, and the mean hemizygous gene proportion in wild progenitor population (*n* = 5, Fig. 1D) was 16.56%, which is slightly higher than that in the cultivar population (15.49%, *n* = 7 with missing long reads of Ries and Vra, Fig. 1D).

### Gene gain and loss during grapevine domestication

**Figure 2.**
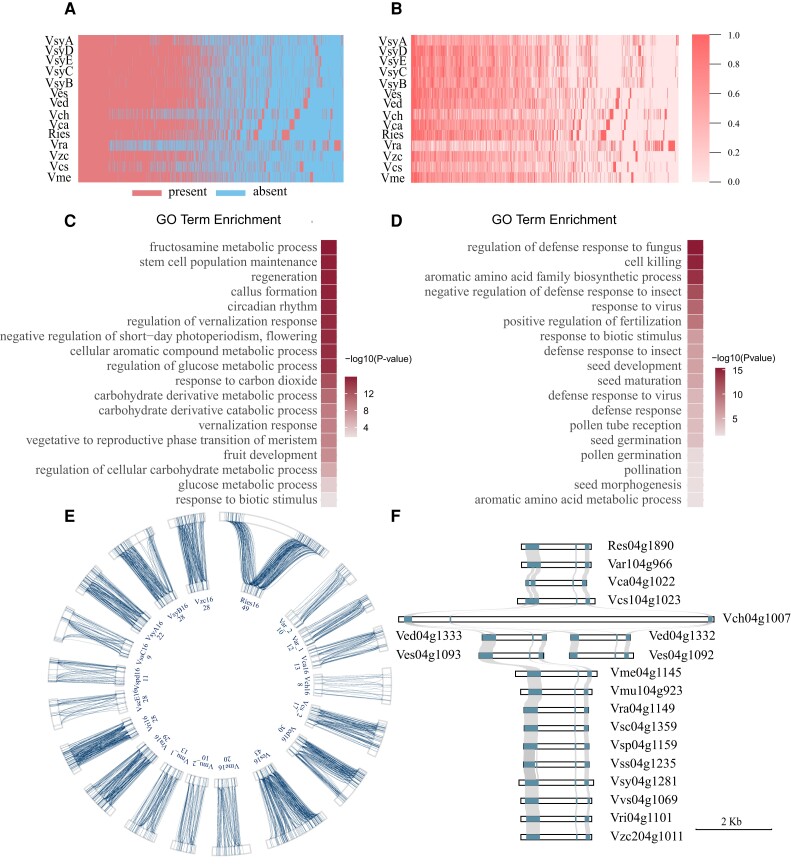
Comparative genomics showed variation of gene contents between different populations, and gene gain and loss during grapevine domestication. **A)** Presence/absence information of pan gene families of wild and cultivars populations. **B)** More/less information of genes in pan gene families of wild and cultivars populations. The color represents the ratio of an accession's gene content to that of the accession with the most genes in that gene family. **C)** GO enrichment result for genes gained by cultivar population. **D)** GO enrichment result for genes lost in cultivar population. **E)** The SVs of the aroma gene cluster. The innermost circle shows the aroma cluster of Vcs while the outermost circle shows the other 16 accessions’ clusters. The lines within the genomes represent the coding sequences (CDS) of aroma genes, and lines between the genomes shows the similarity. **F)** The SVs of the genes that best match the aroma-related gene Vra04g1149 in PN40024. The highlighted squares denote the CDS of these genes.

To study the changes in gene contents during grapevine domestication, we further analyzed the pan-genome based on the combination of wild progenitors and cultivar populations (*n* = 14, Fig. 2, A and B). GO analysis showed that a total of 2,849 gene families (5.56% of 50,750 families), which cultivars have remarkably more members related to wild progenitors (Mann–Whitney–Wilcoxon Test, *P*-value < 0.05) or missed (lost all members) in wild progenitors, were mainly enriched in the biological process related to fruit development, carbohydrate derivative metabolic process, and callus formation process which involve in a vital step of grapevine cutting (Jaleta 2019; Hussein et al. 2021) (Fig. 2C). Whereas, the wild population has more genes in 1,337 families (2.61%) mainly enriched in defense-related process, seed development, and pollen-related function (Fig. 2D). These results are consistent with the fact that most of the cultivated grape are propagated clonally, while most of the wild grape are propagated sexually with seeds. Besides, the genes which function mainly enriched in fruit and carbohydrate derivative metabolic process may reflect the artificial selection on the fruit size and sugar content during domestication (This et al. 2006; Zhou et al. 2017). Interestingly, we also noted that some GO terms, such as aromatic-related process and response to biotic stimulus activity, present in not only the cultivar-more gene families set, but also the wild-more gene families set (Fig. 2, C and D). These may reflect divergent mechanisms of the biological processes in wild relatives and cultivars.

Within the gene families that have remarkable member change associated with grape domestication, we explored the contents of aroma-related genes, which have been of great interest in viticulture and enology. On the basis of aroma genes found in Vcs (Zhou et al. 2017), we searched their homologous genes within other accessions to determine aroma genes in the pan-genome. We notice the dramatic changes in gene numbers in the cultivar and wild progenitor populations (Supplementary Table S4). In chromosome 16 of Vcs, we showed an example of a locus which contains 30 tightly distributed aroma related genes within 500 kb. This locus was defined as an aroma gene cluster, and was further mapped to other accessions to find the homologous region (Supplementary Table S5). Collinearity analysis of this cluster showed there were numerous SVs (Fig. 2, E and F). The aroma-related compound in grapevine has high diversity between different species or cultivars (Ilc et al. 2016; Rahman et al. 2022). Consistent with functional characterization of cultivar-specific SVs in *VviTPS-a*, which play a major role on gene function and floral sesquiterpene profile (Smit et al. 2019), we also detected SVs in aroma-related gene cluster, which may have been selected during domestication. In summary, our results regarding the SVs in the aroma-related gene cluster and the dramatic variation in gene numbers may reveal the potential molecular mechanism underlying the diversity of grapevine aroma (Lin et al. 2019). This information could also provide the candidate materials for further breeding process.

### Resistance gene analogues in the cultivated and wild grapes

Many efforts have been made on investigating the influence of crop domestication on resistance to biotic and antibiotic stress, and it has been found that the domesticated crops are usually more sensitive to stress compare to their wild ancestors (Córdova-Campos et al. 2012; Ferrero et al. 2020; Zhao et al. 2022). For example, in our study, we found that a gene family consists of the homologous genes of *enhanced disease resistant 2* (EDR2) of *Arabidopsis* (*Arabidopsis thaliana*) was extensively present in wild population but lost in cultivar population (Fig. 2, A and B, Mann–Whitney–Wilcoxon Test, *P*-value < 0.05). This gene was shown to have an important function in response to powdery mildew (Vorwerk et al. 2007).

**Figure 3.**
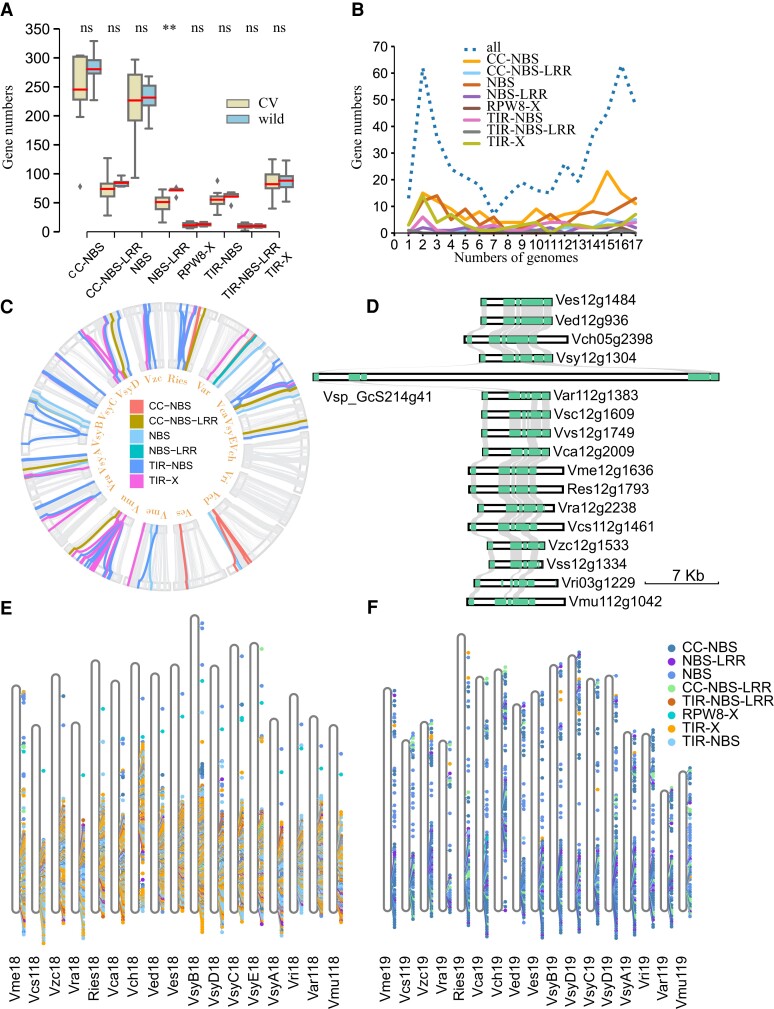
Analyses of the RGAs. **A)** The count of NLR genes in the domesticated population (cv, *n* = 9) and the wild population (*n* = 5). Significance was tested using the Wilcoxon test; ns: no significance, **P ≤ 0.01. Center line, average value; box limits, upper and lower quartiles; whiskers, 1.5× interquartile range; points, outliers. **B)** Distribution of NLR genes of different types. The *x*-axis indicates the number of genomes that possessed such gene families. **C)** Structure of NLR gene cluster within the *MrRUN1/MrRPV1* locus. The innermost circle shows the NLR cluster of Vcs while the outermost circle shows the other 16 genomes. The highlighted lines represent the similarity of CDS from NLR genes of different types. **D)** The SVs of the genes that best match the *MrRPV1* (Vmu112g1042) in Vmu. **E, F)** Distribution of different types of NLR genes on chromosomes 18 (**E**) and 19 (**F**) in the domesticated population (cv) and wild population. CC, TIR, RPW8, NBS, LRR refer to coiled-coil motifs, Toll/interleukin-1 receptor motifs, Resistance to Powdery Mildew 8 domain, nucleotide-binding site, and leucine-rich repeat domain, respectively.

To determine whether the resistance of domesticated grapevine differ from wild progenitor population, we investigated the amount change of resistance gene analogues (RGAs) between wild and cultivated grapes. Firstly, we used the re-annotated protein sequences of 17 genomes to detect nucleotide-binding leucine-rich repeat receptor (NLR) genes, and then classified them into eight types using a previously reported strategy (Van de Weyer et al. 2019). On average, the wild population tend to have more NLR genes than cultivars in eight types of NLR genes (Fig. 3A, Supplementary Table S6). GO enrichment analyses also showed the gene families related to cell killing, which is one of the plant immunities strategies enabled by NLR genes (Yuan et al. 2021), contracted in cultivated population (Fig. 2C). Our results showed that the domesticated grapevine harbored less resistance-related genes compared with wild population, possibly due to the relaxed selection as previously described in tomato (Ferrero et al. 2020).

To investigate the diversity of RGAs in each population, we used long-read sequenced and assembled genomes to construct a graph-based pan-genome (GBPG) for all grapevine (*n* = 17), cultivar (*n* = 9), and wild (*n* = 5) population, respectively. After the trunk genomes were selected, assemblies were subsequently added to the graphs in an iterative manner, with regions of synteny being excluded while sufficiently diverged subsequences (>50 bp) instead augmented the graph with new nodes (“bubbles”). When we compared the GBPG of cultivar population (*n* = 9), wild population (*n* = 5), and all samples (*n* = 17), we found there are 5,420 NLR genes in total in the GBPG for all grapes, while wild and cultivar GBPG have 1,803 and 2,079 NLR genes, respectively (Supplementary Table S7). Considering the bubbles reflect the diversity, we investigated the average diversity contribution (ADC, genes in bubbles divided by the number of accessions) of each accession to NLR genes in each GBPG. In wild GBPG, each wild accession has 284.8 NLR genes’ ADC, while each cultivated accession has 162 ADC of NLR genes in cultivar GBPG. Given that the diversity of cultivar GBPG is higher than wild GBPG (the percentage of genes in bubbles was 68.27% for cultivar GBPG and 29.40% for wild GBPG), the lower NLR genes’ ADC of cultivated accessions highly suggests the homology of themselves, indicating the loss of NLR genes’ diversity during the grape domestication. The counts of trunk NLR genes in GBPG for wild, cultivar and all showed similarity, which indicated the conservative of these trunk NLR genes.

A particularly interesting subset of NLR genes was paired and clustered. We found that the wild have more gene pairs on average, as well as the proportion of paired NLR genes (Supplementary Table S8). Consistently, the wild also have more NLR gene clusters and clustered NLR genes on average, as well as the proportion of clustered NLR genes (Supplementary Table S8).

To further analyze the differences of NLR genes between cultivated and wild grapes, we further performed Pan-NLRome analyses, which use NLR genes to construct Pan-NLR gene families (Van de Weyer et al. 2019). Our results showed that most of the NLR genes showed specificity, indicating a dramatic variation between accessions (Fig. 3B). The same pattern has been found in many other organisms, such as the *Brassica* genus (Zhang et al. 2021b) and maize (Thatcher et al. 2023). We also constructed the NLR gene maps for all 17 accessions (Fig. 3, E and F, Supplementary Fig. S9). NLR genes showed a different distribution on each chromosome. Altogether, our analysis showed a contraction as well as a dramatic diversity of NLR genes during grapevine domestication.

Specifically, downy mildew (*Plasmopara viticola*) can cause serious oomycete disease, which is a major threat to viticulture. Previous studies on Vmu have reported the resistance to *Uncinula necator*/resistance to *P. viticola* (*MrRUN1/MrRPV1*) locus (Feechan et al. 2013) and resistance to *P. viticola* (*MrRPV1*) gene (Qu et al. 2021), which played an important role in grape downy mildew resistance. We mapped the NLR gene clusters of *MrRUN1/MrRPV1* locus on Vcs, and further aligned the same regions on other accessions based on the collinearity (Fig. 3C). In general, cultivars have fewer NLR genes than both wild relatives and Vmu in this region (Fig. 3C). Genes with the highest BlastP alignment scores to *MrRPV1* also harbor frequent SVs in the 16 accessions (Fig. 3D), which may lead to diverse resistance to downy mildew due to the sequence diversity caused by SVs (Foria et al. 2020). The NLRs content changes and SVs between cultivar and wild grapevine found in our work will provide a list of candidates for further functional validation and guidance for breeding process (Foria et al. 2020; Warschefsky and Rieseberg 2021).

## Discussion

Understanding the population genetics of crop domestication is crucial for developing genomic strategies in crop breeding (Gaut et al. 2018; Zhang et al. 2021a). Previous investigations have primarily focused on small variants including SNPs and Indels and SSRs. However, the importance of SVs that potentially have large effects on agronomic traits and biotic and abiotic resistance were ignored for decades, mainly due to technological limitations in sequencing and algorithms detecting (Gaut et al. 2018; Zhou et al. 2019a). Such structural changes can directly lead to the gain or loss of regulatory elements and genes during crop domestication. In grapevine, the domestication process has been investigated with SNP-array (Myles et al. 2011), short-read resequencing data (Zhou et al. 2017; Liang et al. 2019; Zhou et al. 2019a; Freitas et al. 2021; Magris et al. 2021). However, population-scale long-read-based analyses have not yet been conducted. In this study, we apply a common pipeline to assemble and annotate the genomes for representative accessions of wild progenitors, cultivars and relatives of *V. vinifera* using long reads. Comparative genomic and pan-genome analyses were subsequently conducted to study the dramatic variation of the gene content, which revealed the genes gain and loss during grapevine domestication, especially those related to aroma and disease resistance. Our results demonstrate that the graph-based pan-genome could serve as a reference for grapevine genetics, genomics and evolutionary studies. The variations in gene content resulting from large SVs have major impacts on agronomic traits and biotic and abiotic resistances in grapevine and therefore could be used in genetic analyses and breeding of grapevine.

### Gene gain and loss during domestication

We found dramatic variation in gene contents between the two genome copies within each diploid assembly, as well as among accessions within wild progenitor, cultivated grapes and *Vitis* populations, and between wild progenitor and cultivated populations. We observed that only 7% of the gene families shared between all the 16 Vitis samples while 8% of them were specific to each sample. A total of 2,849 gene families which enriched in the biological process related to fruit development, carbohydrate derivative metabolic process and callus formation process gained while 1,337 gene families enriched in defense-related process, seed development and pollen-related function lost in the cultivar population compared with the wild progenitor population, suggesting tremendous changes in gene content associated with grapevine domestication. Overall, our findings highlight the importance of studying the changes of gene contents that occur during domestication and their potential impacts on agronomic traits and resistance to biotic and abiotic stresses in grapevine.

Domestication has shaped many phenotypic traits of grapevine, including the size of berry and cluster size, the berry color, the aroma, and the transition of mating system from dioecy to hermaphrodism (This et al. 2006; Zhou et al. 2017). Previous research investigating QTL regions underlying sex determination and berry color have highlight the importance of SVs and the variation of gene content (Zhou et al. 2019b; Massonnet et al. 2020). Our comparative genomic analyses revealed extensive differences in gene content between the cultivar population and the wild progenitor population. We identified genes that were gained and lost genes in the cultivar population potentially contributing to the development of beneficial agronomic traits. These findings suggest that the domestication of grapevine has had a major impact on the genetic makeup of the plant, resulting in the origin of beneficial agronomic traits.

The domesticated grapes are highly valued for their flavor-rich in winemaking and fresh-eaten. Interestingly, we found a dramatic variation in gene contents related to aroma (Fig. 2). Our findings suggest that combining or manipulating cultivar-specific aroma-related genes could be an effective strategy for aroma breeding in grapevine. However, viticulture has been heavily impacted by pests and diseases, as many cultivars have lost resistance genes during domestication (Fig. 3A). The introduction of resistance genes from grape wild relatives could potentially increase the resistance to various disease. Alternatively, wild *Vitis* with disease resistance could be used as rootstocks and grafted with cultivars to confer resistance (Zhong et al. 2022). These approaches could help mitigate the impact of pests and diseases on grapevine cultivation.

### Comparative genomic analyses capture most of the genomic diversity

In the Chardonnay genome, large SVs affected longer genomic regions than small variants including indels and SNPs between the haploid assemblies (Zhou et al. 2019a). We found ∼15% hemizygous genes within each diploid *Vitis* genomes (Fig. 1D), which suggested that more than 10% of the genes are missing in the PN40024 reference genome (Jaillon et al. 2007; Shi et al. 2023). At the same time, we found only ∼7% of gene families are shared while ∼8% are private to each accession, indicating that we are losing great information using the linear genome from one accession as a reference genome. A general estimate of 60% of the plant trait variations are caused by SVs (Gaut et al. 2018). TEs constitute the majority of angiosperm DNA and contribute a lot to the genomic diversity and SVs, which may lead to several trait variation (Tenaillon et al. 2010; Lisch 2013). In grapes, the berry color variation is associated with TE variation and big inversions (Kobayashi et al. 2004; Carbonell-Bejerano et al. 2017; Zhou et al. 2019a) and the sex determination were determined by the presence and absence of genes or regulatory elements during multiple recombination events (Zhou et al. 2019a, 2019b; Massonnet et al. 2020; Zou et al. 2021). On many occasions, the casual genes of important traits in grapevine were not present in the PN40024 reference genome (Shi et al. 2023).

The pan-genome combined presence/absence variations of many divergent genomes could capture most of the genomic diversity and reduce reference biases in genetic, genomic and evolutionary studies. In tomato, the pan-genome reference reduced reference biases and increased the mapping efficiency compared to the single linear reference genome (Zhou et al. 2022). At the time, the missing heritability of complex agronomic traits could be hidden in the SVs ignored by previous quantitative studies because SVs are mostly unlinked with SNPs (Gaut et al. 2018; Zhou et al. 2019a; Kou et al. 2020). Using of graph-based pan-genome reference and adding SVs have greatly increased the genetic estimates of heritability in tomato metabolic traits (Zhou et al. 2022). We believe our work will greatly improve the studies in grapevine genetics, genomics and evolution, and breeding.

## Conclusion

In conclusion, by utilizing comparative genomic analyses from population level, our result revealed the dramatic variation in gene contents, such as aroma gene clusters and disease-resistance gene clusters caused by SVs. The gain and loss of genes in domesticated grapevine population reveals the possible transition from sexual reproduction to clonal propagation during domestication processes. The gene loss and gain would facilitate the functional genomics and breeding researches in grapevine.

## Materials and methods

### A general pipeline for genome assembly and annotation using PacBio CLR reads

A total of 17 genomes and their corresponding sequencing reads were downloaded from the public database (Supplementary Table S1). Except for Ries and Vra, we collected PacBio CLR reads of 15 genomes. Of these genomes, five were assembled at the chromosome level, which included PN40024 (*V. vinifera* ssp*. vinifera* cv. Pinot Noir clone PN40024), muscadine (*V. rotundifolia*), riverbank grape (*V. riparia*), Arizona grape (*V. arizonica*), and Cabernet Sauvignon (*V. vinifera* cv. Cabernet Sauvignon) (Supplementary Table S1). The remaining 12 genomes were recovered at the scaffold level (Supplementary Table S1), which were assembled to chromosomes level by Ragtag (Alonge et al. 2022) using Vcs as the reference genome in this study. The 17 genome annotations were updated with the upgrade function of Ragtag (Alonge et al. 2022).

To perform pan-genome annotation of the pipeline, we firstly extracted the CDS of the genes from the published annotations of 17 genomes. Then, we eliminate redundancy using CD-HIT v4.7 (Li and Godzik 2006) with the parameters “-c 0.95 -aS 0.9 -G 0.9 -s 0.9”. Thus, the sequences of >95% identity and <10% length variant were reduced to a single category, and the longest sequence of all categories was used to build the CDS database based on pan-genome theory. The sequences from CDS database were re-mapped to each genome using GMAP v2019-06-10 (Wu and Watanabe 2005) with the parameters “-t 10 -*P* -p 3 −min-identity = 0.9 −f”, and the region with ≥90% identity and coverage were selected as the candidate gene regions. The final gene models were predicted by AUGUSTUS v2.7 (Stanke and Morgenstern 2005) with the parameters “−species = arabidopsis −strand = both −singlestrand = false −genemodel = complete −codingseq = on −sample = 100 −keep_viterbi = true −alternatives-from-sampling = true −minexonintronprob = 0.2 −minmeanexonintronprob = 0.5 −maxtracks = 2”. TE annotation was performed by EDTA (Ou et al. 2019) with the parameters “−sensitive 1 −species others −anno 1”. Both gene annotation and TE annotation were performed with soft-masked or unmasked genomes as the input. Correlation analysis was performed using the R package “performanceAnalytics” (Peterson and Carl 2020) in R 4.2.

### Genome completeness and quality evaluation

The genomes completeness were evaluated using BUSCO v.5.0.0 (Manni et al. 2021) with dataset embryophyta_odb10 and Quast v.2.3 (Gurevich et al. 2013). With Vmu hap1 as the reference, genomes synteny analysis of the other 16 genomes was performed via whole-genome alignment using MUMmer4 (Marçais et al. 2018). The best alignment blocks of each other were selected, and then filtered with strict criterions by the delta-filter function of MUMmer4 with option “−1, -r, -l 1200”. Finally, the filtered datasets were used for the dot plot.

### SNPs calling, phylogenetic analyses, and SV identification

Using Vcs as the reference genome, the available PacBio and Illumina sequencing reads of 16 accessions were mapped using minimap2 v2.17-r941 with the parameters “−MD -ax map-pb” (Li 2018) and BWA v0.7.17-r1198-dirty (Li 2013) with default parameters. The mapped reads were further transformed and ordered by SAMtools v0.1.18 (Danecek et al. 2021). SNPs calling were performed using bcftools mpileup and bcftools call v1.10 (Li 2011). The SNPs were further filtered using vcftools v0.1.13 (Danecek et al. 2011) with the parameters “−remove-indels −minDP 18 −minQ 20 −maf 0.05 −stdout −mac 3 −recode −recode-INFO-all −max-missing 1 −min-alleles 2 −max-alleles 2”. The remaining SNPs were used to construct the phylogeny by FastTree v2.1.11 SSE3 (Price et al. 2010).

We performed the SVs calling by: (1) mapping the reads to their genomes’ primary assemblies following the preceding steps of long-read reads alignment; (2) using Sniffles v1.0.12 (Sedlazeck et al. 2018) to identify the SVs. We treated the insertion/deletion as raw presence/absence variation, and further filtered based on their length (shorter than 50 bp and longer than 1 Mbp were dropped) and reads coverage. The genes surrounded by heterozygous presence/absence variation were picked out as the hemizygote, and were counted by bedtools v2.30.0 (Quinlan and Hall 2010).

### Gene family clustering and GO enrichment

Base on the re-annotated proteins from 17 genomes, we retrieved gene families using Orthofinder v2.5.2 (Emms and Kelly 2019) with default settings. Gene families were divided into 17 types based on the number of genomes that have such gene families. The homologous proteins pairs were recovered using MCScanX (Wang et al. 2012), the selection pressure of each pair was calculated with KaKs_Calculator Toolbox v2.0 (Wang et al. 2010). Boxplot was generated using R package “ggplot2” (Wickham 2016) in R 4.2.

We employed the Mann–Whitney U test to test whether there were significant differences of gene family numbers between populations (*P* < 0.05). Gene Ontology annotation was performed in Uniprot (UniProt 2023) by blastp (Camacho et al. 2009). The enrichment test was performed by TBtools (Chen et al. 2020), and GO terms with *P*-value <0.05 were defined as enrichment.

### Aroma-related genes and gene clusters

To define aroma genes of the graph pan-genome, we first recovered the aroma gene lists based on PN40024_v1.0 genome annotation (Zhou et al. 2017). Fifty-eight aroma genes from PN40024_v1.0 were obtained. Then, we extracted CDS sequences of these genes, mapped to gene sequences of Vcs with MUMmer4. The genes with high identity and complete CDS were defined as presumptive aroma genes, then the CDSs of these genes were used as the reference to search the other 16 genomes using the same process.

### NLR gene identification and classification

RGAs are a set of genes that provide immunity for plants to defense various pathogens. Re-annotated proteins of the surveyed 17 genomes were scanned using interproscan v5.51–85.0 (Jones et al. 2014; Blum et al. 2021) to identify the domains. Similar to the pan-NLRome of Arabidopsis (*A. thaliana*) (Van de Weyer et al. 2019), we defined as NLR genes those that contained at least a central nucleotide-binding domain shared by Apaf-1, resistance proteins, and CED4 (NB-ARC), a Toll/interleukin-1 receptor (TIR), or a RPW8-like coiled coil (RPW8) domain. Proteins containing PF00931 (NB-ARC), PF01582 (TIR), and PF05659 (RPW8) domains were selected, and then divided into eight protein classes: CC-NBS, CC-NBS-LRR, NBS, NBS-LRR, RPW8-X, TIR-NBS, TIR-NBS-LRR and TIR-X (Van de Weyer et al. 2019). Later, we identified the conserved domains of the eight protein categories using Multiple EM for Motif Elicitation (MEME) (Bailey et al. 2015) analysis with the flags “-mod anr -nmotifs 20”. The corresponding MEME results of each category were treated with Find Individual Motif Occurrences (FIMO) v.5.3.0 (Grant et al. 2011) on each protein class using the corresponding MEME results. Proteins with at least five conserved domains were kept and used for downstream analysis.

For the diversity analyses of NLRs, we first used minigraph (Li et al. 2020) to add 14 assembled genomes sequenced with third-generation sequencing reads to the reference genome *V. vinifera* cv. Cabernet Sauvignon, consolidating all the genomes into a graphic format. Fifteen annotated sequences are then mapped onto the pan-genome using minigraph. The annotated gene of the reference genome is taken when annotated genes from other genomes share 90% of the gene similarity and unity with *V. vinifera* cv. Cabernet Sauvignon. We chose the longest annotated one in multiple additions. For genes not mapped on the backbone of the map, we used CD-HIT with parameter “-l 0.9” to remove the redundant genes and only retained the genes with the minimum distance from SL5.0. The gene sets mapped to the trunk were then merged and the redundant genes were removed using a CD-HIT with parameter “-l 0.9”. Finally, we used the minigraph -c lr parameter to identify the trunk and bubble annotations. The GBPG of wild and cultivar population were built using the same pipeline, with VsyA and Ves chose to be the trunk, respectively.

Similar to previously reported strategy (Van de Weyer et al. 2019), pan-NLRome analysis were performed by clustering the NLRs with orthofinder v2.5.2. We used RIdeogram (Hao et al. 2020) to visualize the distribution of each NLR genes category across chromosomes. Paired NLR genes were defined as two NLR genes which interval less than two non-NLR genes between them. If more than three genes “paired” to each other, these genes were defined as a cluster.

### *MrRUN1/MrRPV1* locus analyses

To perform *MrRUN1/MrRPV1* locus analyses, we downloaded gene and CDS sequences of *MrRGA1* (JQ904631), *MrRGA2* (JQ904632), *MrRGA4* (JQ904633), *MrRGA8* (*MrRPV1*, JQ90464), *MrRGA9* (JQ904635), *MrRGA10* (*MrRUN1*, JQ904636) and *MrRGA11* (JQ904637) from NCBI. *MrRUN1/MrRPV1* locus were previously defined in *V. rotundifolia* (Feechan et al. 2013). These sequences were mapped to Vmu chromosome 12 using blastn (Camacho et al. 2009). We defined the best colinear regions as the NLR clusters of *MrRUN1/MrRPV1* locus. The *MrRUN1/MrRPV1* NLR cluster of Vcs was delimited by MUMmer4 based on the collinearity with the *MrRUN1/MrRPV1* locus of Vmu (Feechan et al. 2013) as the reference, and *MrRUN1/MrRPV1* NLR cluster of Vcs was further used as the reference to delimited NLR clusters of other 15 genomes. *MrRPV1* was used as the reference to search genes on chromosome 12 in the other accessions by blastp (Camacho et al. 2009), and the best matches were presumptive *RPV1* genes.

### Accession numbers

Genome sequencing data used in this article are available in the NCBI SRA database (PRJNA593045, PRJNA550461, PRJNA517468, PRJNA527006, PRJNA512170, PRJNA316730) and ENA (PRJEB37020). Sequences of NLR genes in *MrRUN1/MrRPV1* locus are available in NCBI: *MrRGA1* (JQ904631), *MrRGA2* (JQ904632), *MrRGA4* (JQ904633), *MrRGA8* (*MrRPV1*, JQ90464), *MrRGA9* (JQ904635), *MrRGA10* (*MrRUN1*, JQ904636), and *MrRGA11* (JQ904637).

## Supplementary Material

kiae039_Supplementary_Data

## Data Availability

The assemblies, the pan-genome and their annotation have been deposited to zenodo: https://doi.org/10.5281/zenodo.7880095.

## Supplementary data

The following materials are available in the online version of this article.

**Supplementary Figure S1.** BUSCO evaluation of 17 genomes.

**Supplementary Figure S2.** Genomic collinearity analysis of 19 chromosomes.

**Supplementary Figure S3.** Correlation coefficients and *P*-values for comparisons between TE size of different families and genome size in 17 grapevines.

**Supplementary Figure S4.** Genome assemblies and component contribution.

**Supplementary Figure S5.** GO enrichment analysis for core and cloud gene set.

**Supplementary Figure S6.** Presence/absence and more/less information of gene families within different populations.

**Supplementary Figure S7.** GO enrichment analysis for cultivar population.

**Supplementary Figure S8.** Comparison of gene families in different haplotypes of Vmu, Var, and Vcs.

**Supplementary Figure S9.** The distribution of NLR genes in each chromosome for the 17 surveyed accessions.

**Supplementary Table S1.** The data sources of samples.

**Supplementary Table S2.** Total genome length and scaffold N50 evaluation of the surveyed 17 genomes.

**Supplementary Table S3.** TE components of the surveyed 17 genomes.

**Supplementary Table S4.** The map quality of the best match aroma genes in each accession.

**Supplementary Table S5.** The position of homologous regions of PN40024 aroma gene cluster.

**Supplementary Table S6.** The counts of NLR genes in each category.

**Supplementary Table S7.** The counts of NLR genes in GBPG.

**Supplementary Table S8.** The counts of paired or clustered NLR genes.
