# Supplementary material for: Population comparative genomics discovers gene gain and loss during grapevine domestication
Source: Plant Physiol. 2024 Jan 29;195(2):1401–13. doi: 10.1093/plphys/kiae039 (PMC11142336; doi:10.1093/plphys/kiae039)
Supplement: kiae039_Supplementary_Data [file kiae039_supplementary_data.pdf]

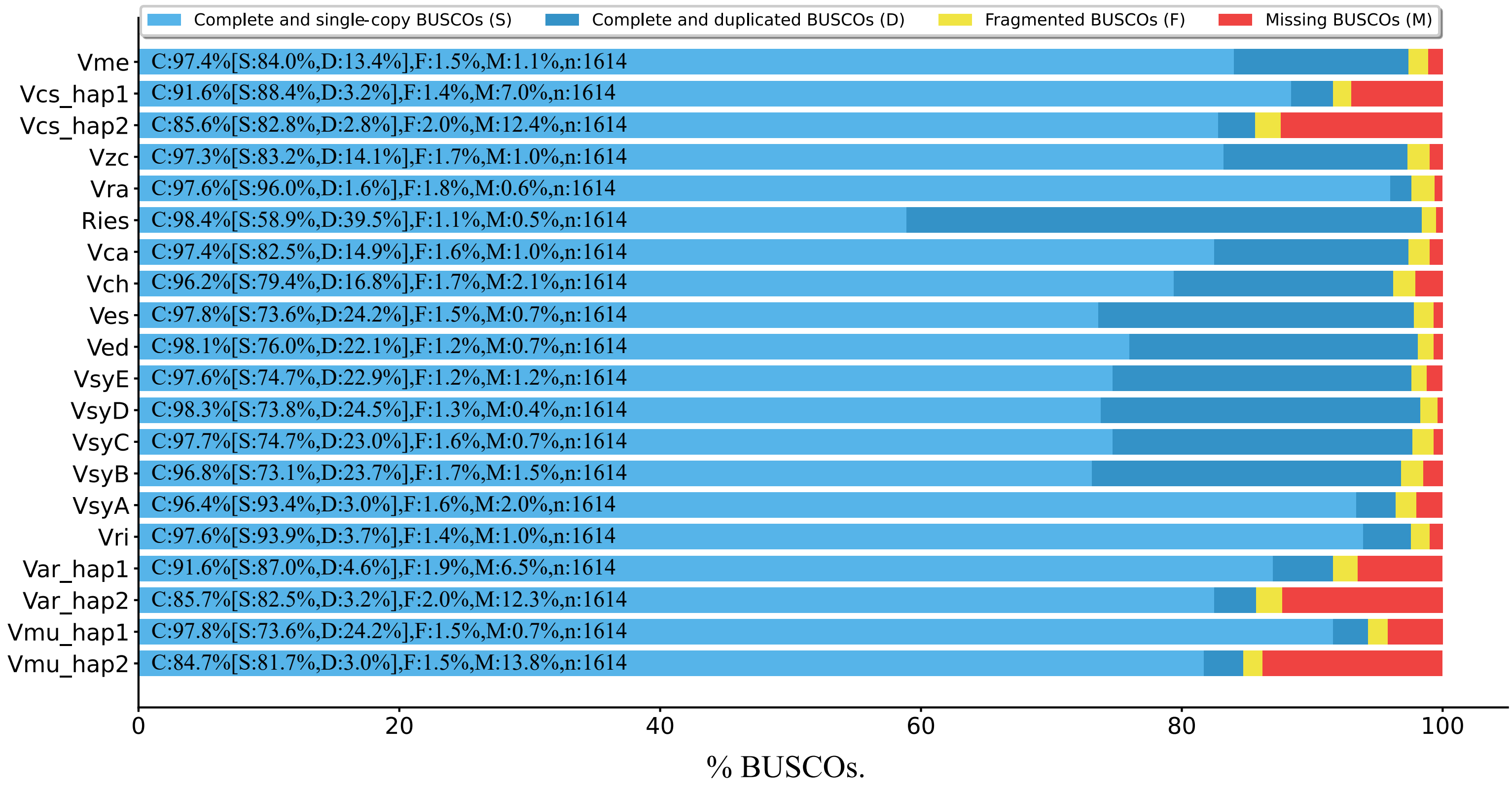

Supplemental Figure S1. BUSCO evaluation of 17 genomes.

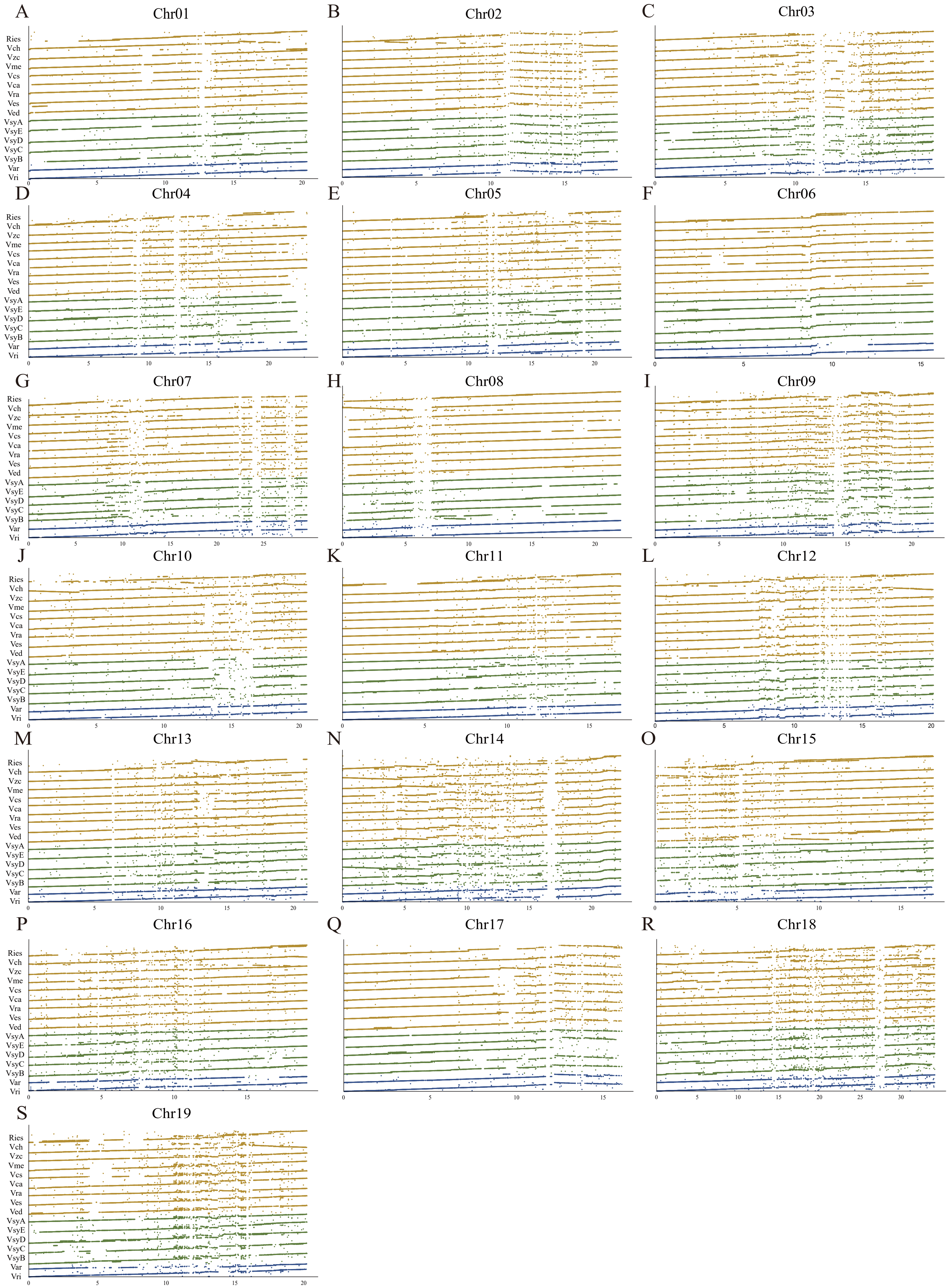

Supplemental Figure S2. Genomic collinearity analysis of 19 chromosomes. Chromosomes 1-19 (A-S), the *x*-axes refer to the chromosome position of Vmu (Mbp), the *y*-axes refer to the chromosome position of 16 accessions, dots refer to the collinearity.

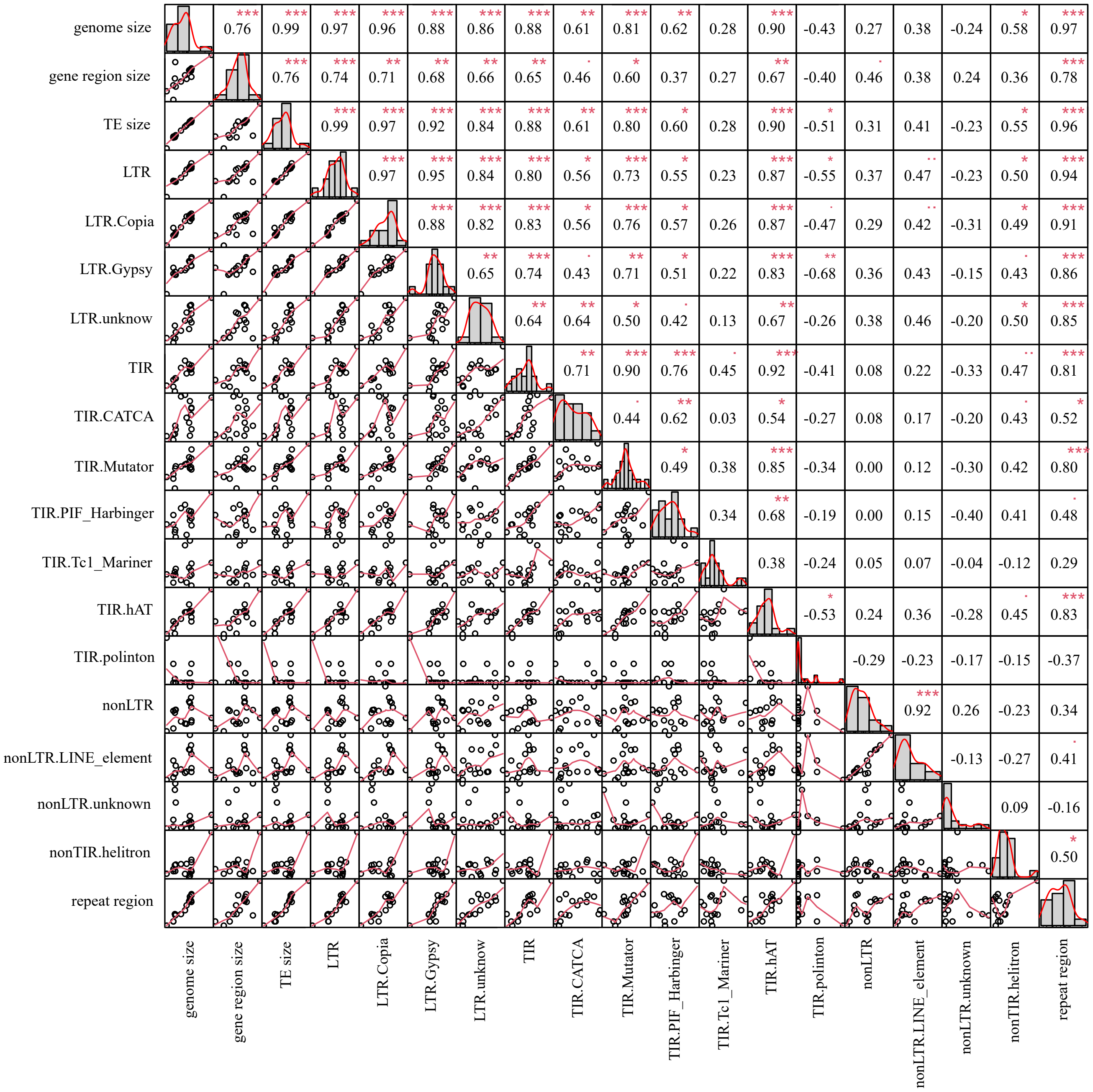

Supplemental Figure S3. Correlation coefficients and *P*-values for comparisons between TE size of different families and genome size in 17 grapevines. The lower left part of the diagonal diagram shows the distribution of data. The upper right part of the diagonal diagram is the reflection of the correlation among each TE family (Pearson correlation coefficient, * indicate a *P*-value < 0.1, ** indicate a *P*-value < 0.05, *** indicate a *P*-value < 0.01). Diagonal showes the distribution spectrum of data.

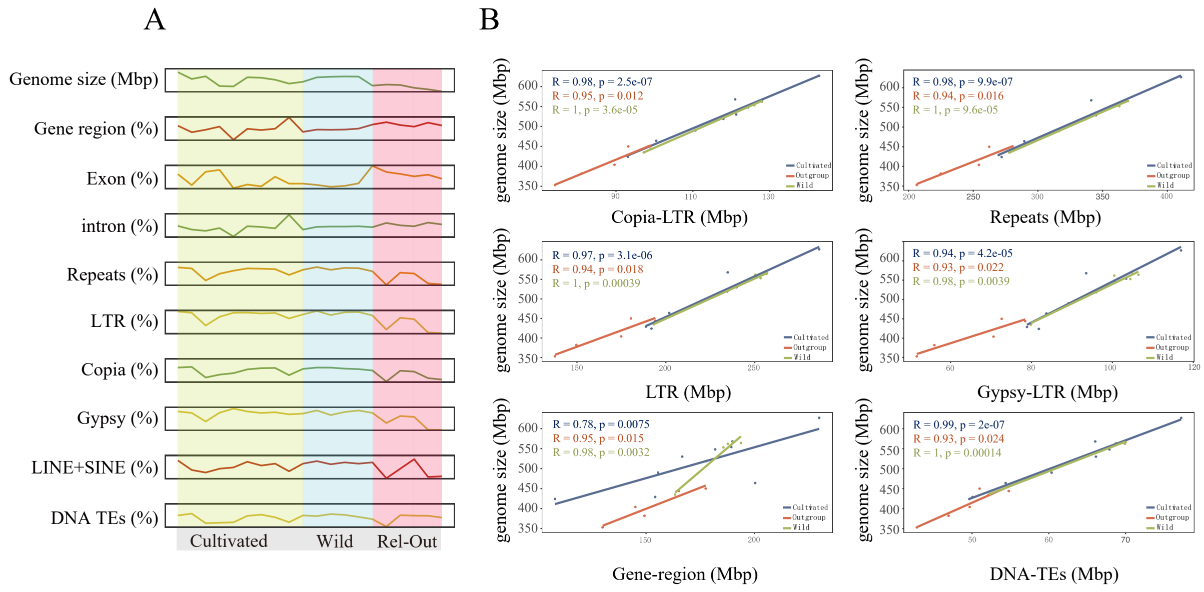

Supplemental Figure S4. Genome assemblies and component contribution.

(A) Landscape of genome size and percentage of transposable element (TEs) within it for different populations, including the percentage of genomic regions, exons, introns, repeats, LTRs, Gypsy LTRs, Copia LTRs, SINEs + LINEs and DNATEs of different lengths in the genome. Rel-Out refer to the wild relative population and outgroup (Vmu).

(B) Pearson correlation coefficients for comparison of genome size with total length of annotated regions, total length of TEs, total length of DNATEs, and total length of LTRs across populations. Colored dots and lines indicate data from different populations. Relatives are classified into outgroups.

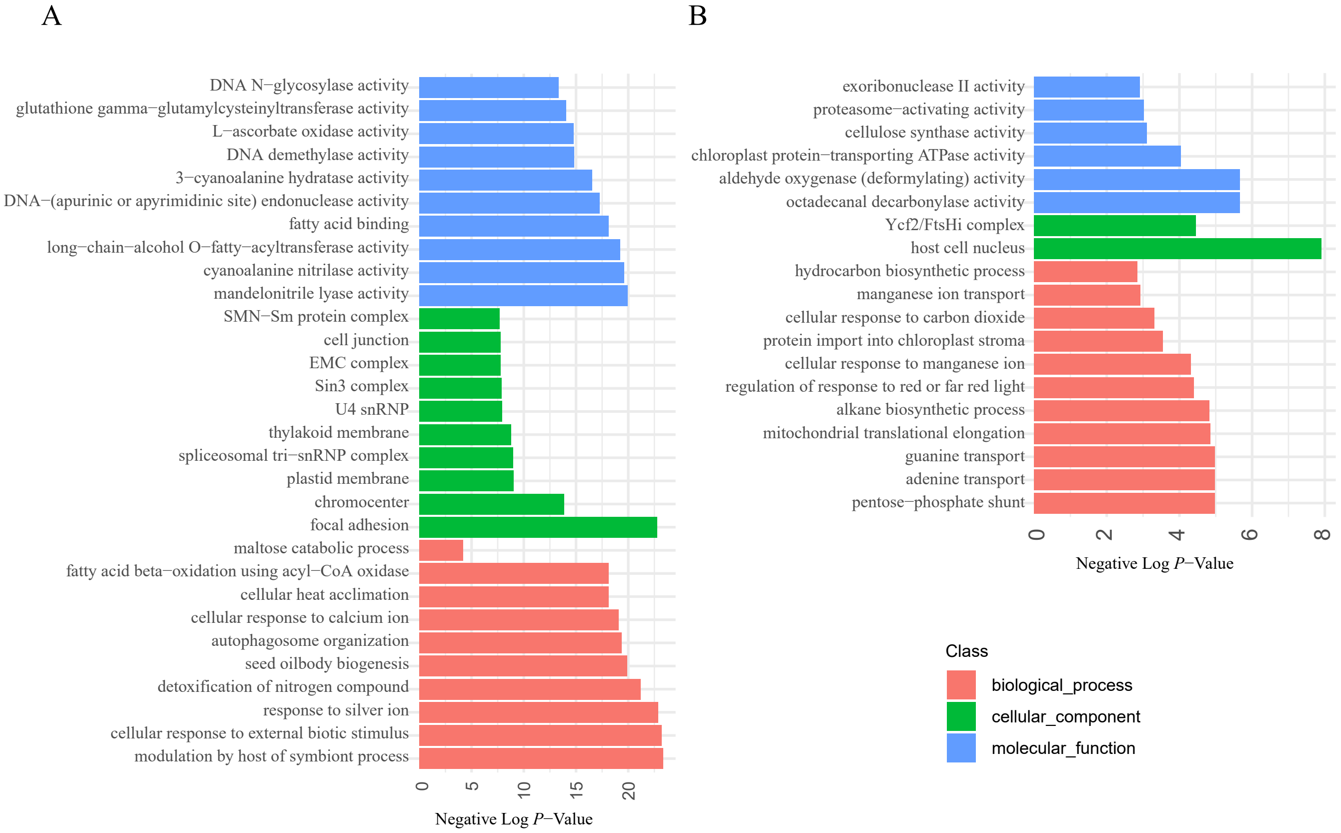

Supplemental Figure S5. GO enrichment analysis for core and cloud gene set. A, GO enrichment result for genes in core gene families. B, GO enrichment result for genes in cloud gene families.

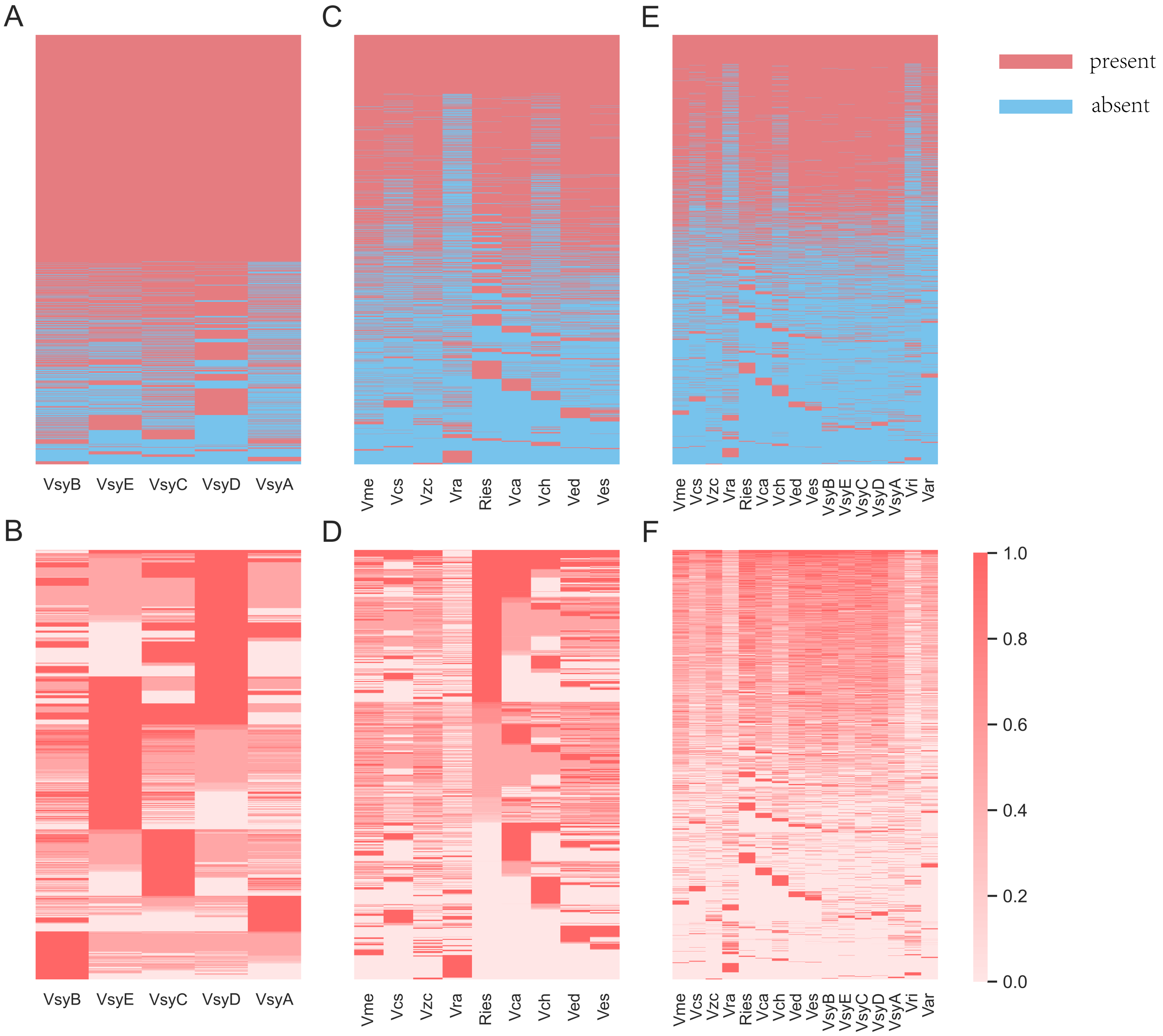

Supplemental Figure S6. Presence/absence and more/less information of gene families within different populations. A, C, E, Presence/absence information of wild population (A), cultivar population (C) and *Vitis* (E). B, D, F, More/less information of wild population (B), cultivar population (D) and *Vitis* (F). The color represents the ratio of an accession's gene content to that of the accession with the most genes in that gene family. The ***y***-axes refer to different orthogroups classfied by orthofinder.

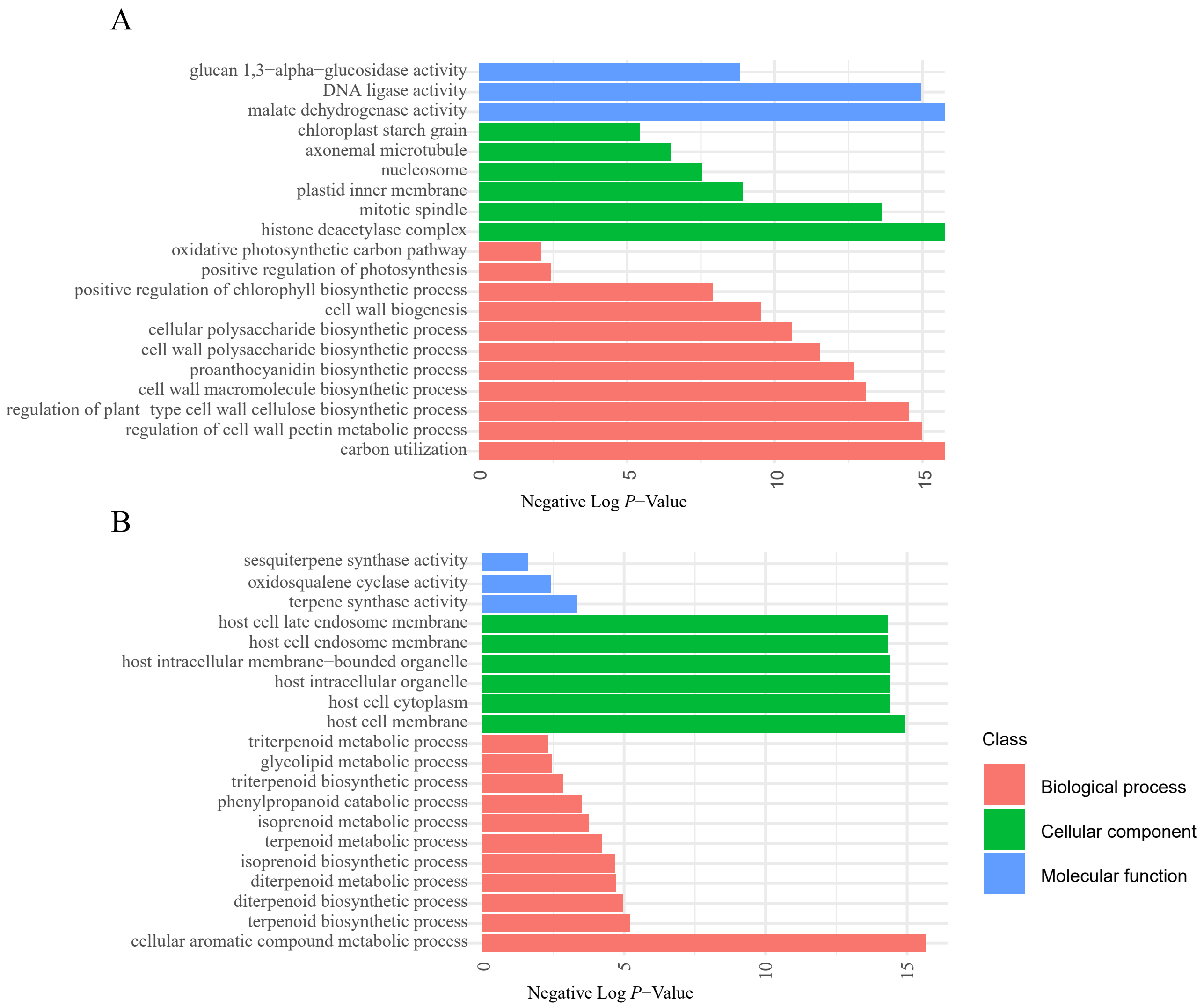

Supplemental Figure S7. GO enrichment analysis for cultivar population. A, GO enrichment result for genes in the families that owned by all cultivars. B, GO enrichment result for genes in the families that owned by only one accession. GO terms with *P*-value ≤ 0.05 were defined as enrichment.

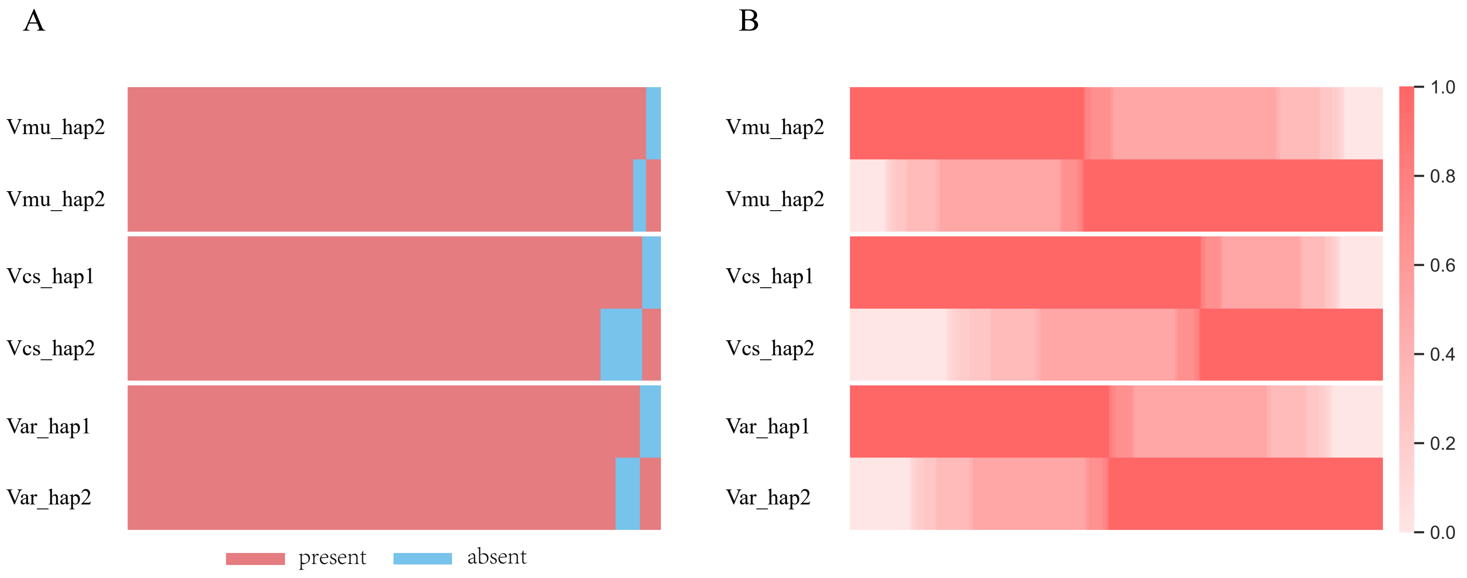

Supplemental Figure S8. Comparison of gene families in different haplotypes of Vmu, Var and Vcs. The *x*-axes refer to different orthogroups classfied by orthofinder. A, Presence/absence information of different haplotypes. B, More/-less information of different haplotypes. The color represents the ratio of an accession's gene content to that of the accession with the most genes in that gene family. The *x*-axes refer to different orthogroups classfied by orthofinder.

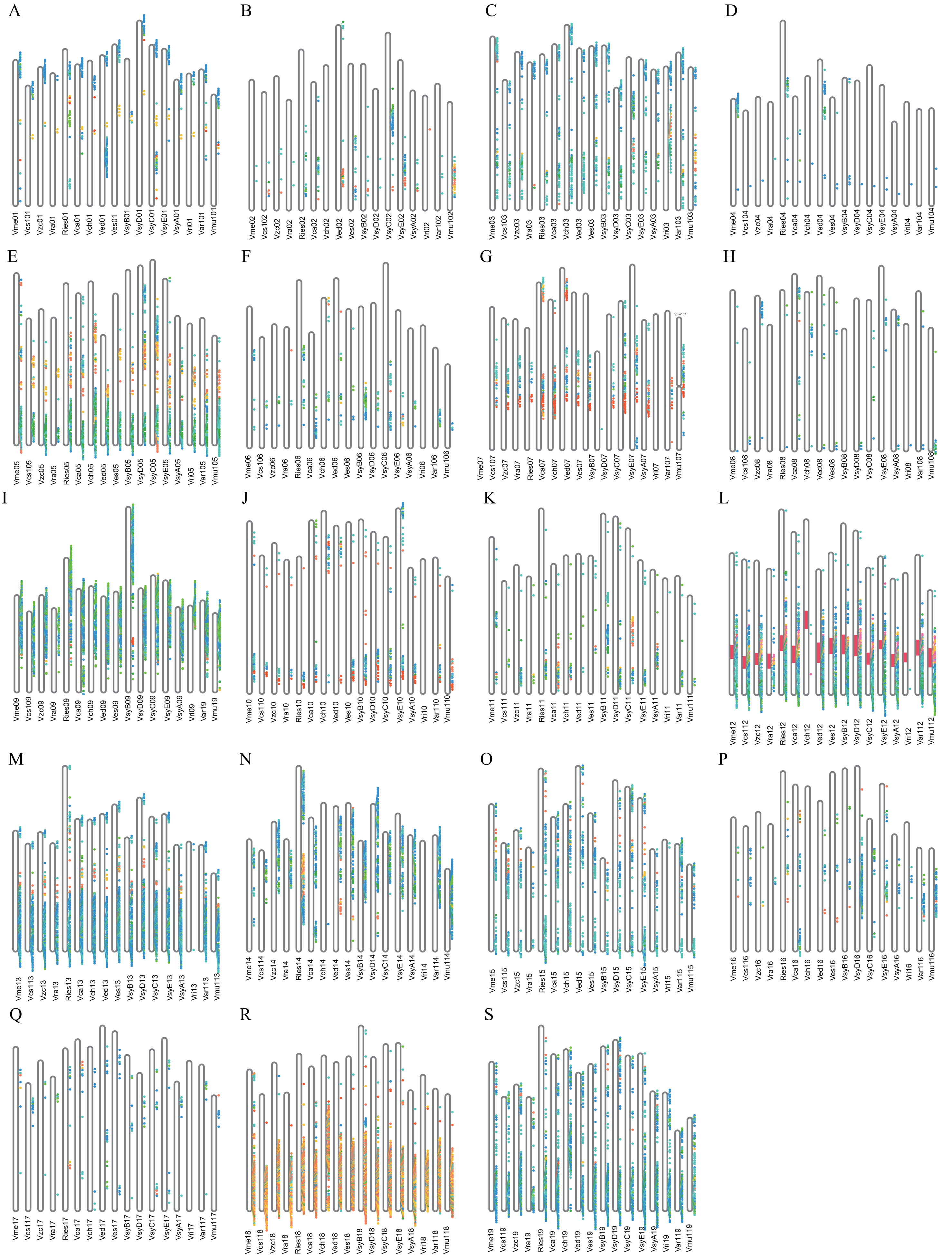

Supplemental Figure S9. The distribution of NLR genes in each chromosome for the 17 surveyed accessions. A-S, the distribution of NLR genes in chromosome 1-19.

Supplemental Table S1. The data sources of samples.

Species	Abbreviation	reference
Vitis vinifera cv. Black Corinth Seedless	Ves	(Massonnet et al., 2020)
Vitis vinifera cv. Black Corinth seeded	Ved	(Massonnet et al., 2020)
Vitis vinifera cv. Cabernet Sauvignon	Vcs	(Massonnet et al., 2020)
Vitis vinifera cv. Carménère	Vca	(Minio et al., 2019)
Vitis vinifera cv. Chardonnay	Vch	(Zhou et al., 2019)
Vitis vinifera cv. Merlot	Vme	(Massonnet et al., 2020)
Vitis vinifera cv. Riesling	Ries	(Zou et al., 2021)
Vitis vinifera cv. Zinfandel	Vzc	(Vondras et al., 2019)
PN40024	Vra	(Shi et al., 2023)
Vitis vinifera ssp. *sylvestris*	VsyA	(Badouin et al., 2020)
Vitis vinifera ssp. *sylvestris* DVIT3351.27	VsyB	(Massonnet et al., 2020)
Vitis vinifera ssp. *sylvestris* DVIT3603.07	VsyC	(Massonnet et al., 2020)
Vitis vinifera ssp. *sylvestris* DVIT3603.16	VsyD	(Massonnet et al., 2020)
Vitis vinifera ssp. *sylvestris* O34-16	VsyE	(Massonnet et al., 2020)
Vitis riparia	Vri	(Girollet et al., 2019)
Vitis arizonica	Var	(Massonnet et al., 2020)
Muscadinia rotundifolia 'Trayshed'	Vmu	(Massonnet et al., 2020)

Badouin H, Velt A, Gindraud F, Flutre T, Dumas V, et al. (2020) The wild grape genome sequence provides insights into the transition from dioecy to hermaphroditism during grape domestication. Genome Biol **21:** 223

Girollet N, Rubio B, Lopez-Roques C, Valiere S, Ollat N, et al. (2019) De novo phased assembly of the Vitis riparia grape genome. Sci Data **6:** 127

Massonnet M, Cochetel N, Minio A, Vondras AM, Lin J, et al. (2020) The genetic basis of sex determination in grapes. Nat Commun **11:** 2902

Minio A, Massonnet M, Figueroa-Balderas R, Castro A, Cantu D (2019) Diploid Genome Assembly of the Wine Grape Carmenere. G3 (Bethesda) **9:** 1331-1337

Shi X, Cao S, Wang X, Huang S, Wang Y, et al. (2023) The complete reference genome for grapevine (Vitis vinifera L.) genetics and breeding. Horticulture Research

Vondras AM, Minio A, Blanco-Ulate B, Figueroa-Balderas R, Penn MA, et al. (2019) The genomic diversification of grapevine clones. BMC Genomics **20:** 972

Zhou Y, Minio A, Massonnet M, Solares E, Lv Y, et al. (2019) The population genetics of structural variants in grapevine domestication. Nat Plants **5:** 965-979

Zou C, Massonnet M, Minio A, Patel S, Llaca V, et al. (2021) Multiple independent recombinations led to hermaphroditism in grapevine. Proc Natl Acad Sci USA **118**

Supplemental Table S2. Total genome length and scaffold N50 evaluation of the surveyed 17 genomes. The length are showed in Mb.

	total	Longest	N10	N20	N30	N40	N50	N60	N70	N80	N90
Vmu_hap1	400.45	34.10	23.20	22.04	22.00	21.00	20.34	20.16	19.08	17.00	15.70
Vmu_hap2	369.99	26.29	24.02	22.44	22.36	20.38	18.58	17.25	17.06	16.49	14.26
Var_hap1	465.55	35.71	31.36	31.16	28.51	25.58	25.10	23.89	22.08	20.36	19.19
Var_hap2	422.78	35.82	29.03	28.36	26.26	23.91	22.83	20.73	20.53	18.58	16.12
Vri	471.20	39.74	30.41	29.50	25.68	24.45	23.92	23.49	22.71	20.88	19.82
VsyA	455.94	2.54	0.57	0.39	0.29	0.23	0.17	0.13	0.09	0.06	0.03
VsyB	579.85	17.90	4.74	3.69	3.03	2.47	1.78	1.46	1.13	0.82	0.46
VsyC	588.86	7.36	3.90	2.55	1.92	1.50	1.17	0.92	0.66	0.45	0.26
VsyD	590.95	8.51	5.91	5.00	4.19	3.26	2.66	2.27	1.72	1.22	0.64
VsyE	592.60	6.39	3.13	2.10	1.62	1.30	1.00	0.75	0.57	0.39	0.22
Ved	580.12	9.11	6.36	4.23	3.62	2.72	2.31	1.82	1.38	0.90	0.50
Ves	574.06	6.69	3.27	2.54	1.72	1.43	1.11	0.86	0.64	0.44	0.24
Vch	594.72	35.15	34.60	27.81	26.70	25.77	24.53	23.02	20.25	5.31	0.28
Vca	555.51	5.91	3.24	2.14	1.64	1.35	1.04	0.76	0.56	0.37	0.20
Ries	657.40	12.50	7.31	5.12	4.18	3.48	2.97	2.41	1.84	1.37	0.77
Vra	486.21	34.57	30.27	27.39	27.36	24.71	24.27	23.57	23.01	20.70	20.12
Vzc	512.98	7.90	3.20	2.19	1.82	1.37	1.06	0.71	0.47	0.29	0.16
Vcs_hap1	449.79	34.11	29.51	28.97	26.05	24.76	23.18	22.11	21.90	20.76	19.35
Vcs_hap2	444.21	36.59	27.25	26.68	25.54	24.55	24.16	23.05	21.79	20.20	19.41
Vme	543.98	6.32	2.67	1.77	1.29	1.04	0.81	0.64	0.46	0.31	0.19

Supplemental Table S3. TE components of the surveyed 17 genomes.

Class		Ries			Vca			Vch			Vcs		
		primary			primary			primary			primary		
		Count	bpMasked	%masked	Count	bpMasked	%masked	Count	bpMasked	%masked	Count	bpMasked	%masked
**LTR**	Copia	81664	69275532	9.34%	74077	54865936	9.89%	68416	55792044	9.45%	45358	37713293	8.27%
	Gypsy	85268	92879321	12.53%	64917	64264018	11.58%	68070	67679383	11.46%	53583	64878567	14.22%
	unknown	78671	43221986	5.83%	69057	35034602	6.32%	65240	34410937	5.83%	34373	18311459	4.01%
**TIR**	CACTA	48087	19683987	2.66%	33574	16114460	2.90%	36130	17882009	3.03%	29729	13133726	2.88%
	Mutator	77309	30127368	4.06%	56172	21779226	3.93%	61117	23931216	4.05%	44485	17451133	3.83%
	PIF_Harbinger	25949	10376220	1.40%	11202	4551430	0.82%	19333	6798412	1.15%	19926	7247694	1.59%
	Tc1_Mariner	8301	2876052	0.39%	3417	889108	0.16%	4102	1670194	0.28%	3143	1609517	0.35%
	hAT	26123	14168334	1.91%	19713	9440722	1.70%	17923	7509986	1.27%	16126	7523722	1.65%
	polinton	0	0	0	0	0	0	75	28129	0.00%	0	0	0
**nonLTR**	LINE_element	1105	345698	0.05%	871	298957	0.05%	936	289853	0.05%	418	126455	0.03%
	unknown	553	151758	0.02%	20757	8343260	1.50%	303	62957	0.01%	117	21570	0.00%
**nonTIR**	helitron	48279	15820571	2.13%	0	0	0.00%	28574	9305889	1.58%	23731	7101584	1.56%
repeat_region		179503	66470818	8.97%	136021	47472950	8.56%	149969	55210865	9.35%	99678	35061462	7.69%
**total interspersed**		660812	365397645	49.29%	489778	263054669	47.42%	520188	280571874	47.50%	370667	210180182	46.08%

Class		Ved			Ves			Vme			Vmu		
		primary			primary			primary			primary		
		Count	bpMasked	%masked	Count	bpMasked	%masked	Count	bpMasked	%masked	Count	bpMasked	%masked
**LTR**	Copia	79717	56416665	9.73%	70264	55167053	9.62%	65251	50961332	9.38%	39933	28569006	7.04%
	Gypsy	75866	74248571	12.81%	66471	74856576	13.05%	61804	61221647	11.27%	30347	25174898	6.20%
	unknown	64856	30988724	5.35%	51220	25877446	4.51%	52843	30526933	5.62%	43666	20894393	5.15%
**TIR**	CACTA	34449	12949419	2.23%	39685	15230945	2.65%	48353	20138313	3.71%	37211	12869420	3.17%
	Mutator	59768	22789880	3.93%	60219	24464914	4.26%	53411	21479236	3.95%	41458	16325444	4.02%
	PIF_Harbinger	20219	7112524	1.23%	21668	7829792	1.36%	15873	8642896	1.59%	16831	5978797	1.47%
	Tc1_Mariner	9596	5090836	0.88%	4495	2387281	0.42%	11268	4621810	0.85%	3524	1707483	0.42%
	hAT	22791	9690944	1.67%	22381	11022093	1.92%	19199	9414655	1.73%	12623	4893386	1.21%
	polinton	0	0	0.00%	0	0	0	0	0	0	109	66823	0.02%
**nonLTR**	LINE_element	2456	949598	0.16%	1531	967546	0.17%	592	263098	0.05%	128	53359	0.01%
	unknown	186	43523	0.01%	218	68613	0.01%	408	46896	0.01%	0	0	0
**nonTIR**	helitron	15067	5532306	0.95%	16564	5239253	0.91%	20666	5980751	1.10%	19348	6016584	1.48%
repeat_region		141886	52147267	9.00%	142744	50709644	8.84%	122772	43654411	8.04%	95286	31848945	7.85%
**total interspersed**		526857	277960257	47.95%	497460	273821156	47.73%	472440	256951978	47.31%	340464	154398538	38.05%

Class		Vra			Vri			VsyD			VsyE		
		primary			primary			primary			primary		
		Count	bpMasked	%masked	Count	bpMasked	%masked	Count	bpMasked	%masked	Count	bpMasked	%masked
**LTR**	Copia	47850	39116171	8.32%	55833	38250105	8.81%	76127	57464962	9.73%	65666	52885299	8.94%
	Gypsy	57271	58771633	12.50%	53522	54581206	11.68%	70591	78459580	13.28%	82067	80723922	13.65%
	unknown	43604	20740336	4.41%	46399	27759560	5.94%	71393	36598926	6.20%	63503	29505707	4.99%
**TIR**	CACTA	33496	14699695	3.13%	30227	13256086	2.84%	39025	17745092	3.00%	37342	14087264	2.38%
	Mutator	49027	19806765	4.21%	32041	11619218	2.49%	48724	19417550	3.29%	68824	27669108	4.68%
	PIF_Harbinger	16755	5237645	1.11%	16093	4900445	1.05%	16692	7325833	1.24%	16340	5836609	0.99%
	Tc1_Mariner	3954	1654473	0.35%	6151	1951676	0.42%	1742	579798	0.10%	4954	2788650	0.47%
	hAT	16174	7078887	1.51%	9707	5017941	1.07%	15569	8403656	1.42%	20510	9593929	1.62%
	polinton	0	0	0	21	5754	0.00%	0	0	0	0	0	0.00%
**nonLTR**	LINE_element	557	258820	0.06%	755	237126	0.05%	1525	829879	0.14%	1024	405131	0.07%
	unknown	816	500398	0.11%	652	429617	0.09%	23	10627	0.00%	21	8438	0.00%
**nonTIR**	helitron	24612	6634922	1.41%	27473	8036421	1.72%	25121	8719811	1.48%	22555	6537732	1.11%
repeat_region		107253	39694831	8.44%	117554	40732048	8.72%	137439	50071318	8.48%	160066	56100077	9.49%
**total interspersed**		401369	214194576	45.55%	396428	206777203	44.24%	503971	285627032	48.36%	542872	286141866	48.38%

Class		VsyC			VsyA			Vzc			VsyB		
		primary			primary			primary			primary		
		Count	bpMasked	%masked	Count	bpMasked	%masked	Count	bpMasked	%masked	Count	bpMasked	%masked
**LTR**	Copia	68178	54126239	9.20%	61602	44589532	9.78%	60052	50700783	8.75%	54776	45126705	8.80%
	Gypsy	64476	68036610	11.57%	53348	55856451	12.25%	70349	73323066	12.65%	60204	60740476	11.84%
	unknown	70164	39249470	6.67%	41886	21171847	4.64%	72950	38781938	6.69%	38942	24960146	4.87%
**TIR**	CACTA	36342	16638431	2.83%	29291	12215194	2.68%	44085	19045022	3.29%	44696	19859226	3.87%
	Mutator	48347	20364017	3.46%	45625	15634483	3.43%	54377	19915773	3.44%	49555	20647953	4.03%
	PIF_Harbinger	19396	7736826	1.32%	14264	5936457	1.30%	16616	6507379	1.12%	16916	8172433	1.59%
	Tc1_Mariner	5872	2103059	0.36%	2170	874078	0.19%	4850	1545705	0.27%	2817	1134882	0.22%
	hAT	18782	9697221	1.65%	16532	6179613	1.36%	16172	9317722	1.61%	17784	8056357	1.57%
	polinton	0	0	0	99	28056	0.01%	55	14235	0.00%	0	0	0
**nonLTR**	LINE_element	2086	1092372	0.19%	887	631813	0.14%	2421	1401247	0.24%	1424	368366	0.07%
	unknown	521	72945	0.01%	327	96348	0.02%	466	138865	0.02%	68	11216	0.00%
**nonTIR**	helitron	24659	6868783	1.17%	20569	6607555	1.45%	21791	6023195	1.04%	26236	8283474	1.62%
repeat_region		139102	51113206	8.69%	97833	35041773	7.69%	146639	54133846	9.34%	113873	40696652	7.93%
**total interspersed**		497925	277099179	47.12%	384433	204863200	44.94%	510823	280848776	48.47%	427291	238057886	46.42%

Class		Var		
		primary		
		Count	bpMasked	%masked
**LTR**	Copia	52928	40410208	8.53%
	Gypsy	55810	54192754	11.44%
	unknown	39592	24127263	5.09%
**TIR**	CACTA	30578	14018749	5.09%
	Mutator	44741	20733657	4.38%
	PIF_Harbinger	10592	4253606	0.90%
	Tc1_Mariner	5168	2261175	0.48%
	hAT	15917	7092551	1.50%
	polinton	0	0	0.00%
**nonLTR**	LINE_element	851	478482	0.10%
	unknown	322	287973	0.06%
**nonTIR**	helitron	25352	8183564	1.73%
repeat_region		106158	40279670	8.50%
**total interspersed**		388009	216319652	45.66%

Supplemental Table S4. The map quality of the best match aroma genes in each accession. "1" means genes which have >95% mapping identity and length related to corresponding genes in PN40024 can be found. "2" means genes that were fragmentarily mapped to corresponding genes in PN40024 can be found. “Un” means only fragmentarily matched genes from unplaced scaffolds can be found. "0" means no matching result can be found.

The map quality of the best match aroma genes in each accession

			Cultivar									Wild								Out-Group		
Gene ID(PN40024)	Function	lenth(bp)	Ved	Ves	Vch	Vzc	Vca	Vme	Ries	Vcs1	Vcs2	Vsy	Vvs	Vsp	Vss	Vsc	Vri	Var1	Var1	Vmu1	Vmu2	count
VIT_03s0063g01830	9-hydroperoxidase	8830	0	0	0	0	0	0	2	2	0	0	0	2	2	2	0	0	0	0	0	0
VIT_00s0847g00020	Linalool/(E)-Nerolidol/ (E,E)-Geranyl linalool synthase	2280	2	2	2	2	2	2	2	2	1	2	2	2	2	2	2	2	2	2	2	1
VIT_00s0385g00020	(3s)-Linalool/(E)-Nerolidol synthase	2238	2	1	2	2	2	2	1	1	2	2	2	1	1	2	2	2	2	2	2	5
VIT_00s0324g00050	Furaneol glucosyltransferase	8459	2	2	1	2	2	1	1	2	2	2	2	2	1	1	2	0	2	0	0	5
VIT_13s0064g00810	Carotene cleavage dioxygenase	8755	2	1	1	2	1	1	0	1	2	2	2	2	2	2	0	1	2	0	2	6
VIT_13s0067g00370	(-)-α-Terpineol synthase	2550	1	1	2	2	2	0	2	2	1	2	0	1	1	2	2	2	1	2	2	6
VIT_06s0004g01510	Type II lipoxygenase	6627	2	2	2	2	1	2	2	1	2	1	1	2	1	1	0	2	2	0	0	6
VIT_09s0018g01490	Alcohol dehydrogenase	2071	2	0	1	2	1	1	Un	1	0	1	1	1	0	0	1	2	2	2	2	8
VIT_11s0016g02830	γ-Glutamyltranspeptidase	3649	1	1	1	1	1	2	2	2	0	2	1	2	1	2	2	2	2	2	2	7
VIT_12s0059g01750	S-Adenosyl-L-Met (SAM)-dependent O-methyltransferase	1504	0	2	0	0	2	2	2	2	0	1	2	2	1	1	1	1	1	1	1	8
VIT_05s0020g02130	1-Deoxy-D-xylulose 5-phosphate synthase	5121	1	1	2	2	2	2	2	2	2	2	2	2	2	2	1	1	1	1	1	7
VIT_03s0038g03080	S-Adenosyl-L-Met (SAM)-dependent O-methyltransferase	2739	1	1	2	1	2	1	1	2	0	1	1	0	1	1	1	2	2	0	0	10
VIT_04s0044g01110	Alcohol dehydrogenase	2789	Un	0	1	1	1	Un	1	0	1	Un	1	1	Un	1	1	1	1	1	1	13
VIT_03s0038g03090	S-Adenosyl-L-Met (SAM)-dependent O-methyltransferase	1373	1	1	1	1	1	1	1	1	0	1	1	0	1	1	1	1	1	0	2	15
VIT_04s0079g00690	Glutathione synthase	2285	1	1	1	1	1	1	1	1	1	1	1	1	1	1	0	2	0	0	0	14
VIT_12s0134g00140	Geraniol synthase	1380	1	0	1	1	1	1	1	1	1	1	1	1	1	1	2	1	0	2	2	14
VIT_19s0014g02590	Selinene synthase polymorphic variant	2314	1	1	1	1	1	1	1	2	1	1	2	1	1	1	2	2	2	1	0	13
VIT_12s0028g00920	Glutathione synthase	1399	1	1	1	1	1	2	1	1	1	1	1	1	1	1	1	1	1	1	1	18
VIT_12s0059g01060	13-hydroperoxidase	2866	1	1	1	1	1	1	1	2	1	1	1	1	1	1	1	1	1	0	0	16
VIT_16s0050g01580	Glucosyltransferase	1640	1	1	1	1	1	1	1	1	0	1	1	1	0	1	1	0	0	1	0	14
VIT_02s0087g00910	Carotene cleavage dioxygenase	1896	1	1	1	1	1	1	1	1	Un	1	Un	1	1	1	1	1	1	1	0	16
VIT_02s0087g00930	Carotene cleavage dioxygenase	1934	1	1	1	1	1	1	1	1	Un	1	Un	1	1	1	1	1	1	1	0	16
VIT_01s0127g00740	Enone reductase	2096	1	1	1	1	1	1	1	1	1	1	1	2	1	1	1	1	1	1	Un	17
VIT_12s0059g01790	S-Adenosyl-L-Met (SAM)-dependent O-methyltransferase	1287	1	1	1	1	1	1	1	1	1	1	1	1	1	1	1	1	1	1	2	18
VIT_13s0064g00840	Carotene cleavage dioxygenase	8917	1	1	1	1	1	1	0	1	1	1	1	1	1	1	1	2	1	0	2	15
VIT_19s0015g00110	Sesquiterpene oxidase α-guaiene 2-oxidase	1856	1	1	1	1	1	1	1	0	1	Un	1	1	1	1	1	0	1	1	1	16
VIT_18s0001g06060	Glucosyltransferase	1645	1	1	1	1	1	Un	0	1	1	1	1	1	1	1	1	1	1	1	1	17
VIT_18s0001g15410	Alcohol dehydrogenase	2491	1	1	1	1	1	1	1	1	1	1	1	1	1	1	1	1	0	1	1	18
VIT_18s0001g15450	Alcohol dehydrogenase	2930	1	1	1	1	1	1	1	1	1	1	1	1	1	1	1	1	0	1	1	18
VIT_00s0372g00040	Linalool/(E)-Nerolidol/ (E,E)-Geranyl linalool synthase	2260	1	1	1	1	1	1	1	1	1	1	1	1	1	1	1	1	1	1	1	19
VIT_00s0372g00070	Linalool/(E)-Nerolidol/ (E,E)-Geranyl linalool synthase	2681	1	1	1	1	1	1	1	1	1	1	1	1	1	1	1	1	1	1	1	19
VIT_02s0025g04880	(E)-8-Carboxylinalool synthase	1639	1	1	1	1	1	1	1	1	1	1	1	1	1	1	1	1	1	1	1	19
VIT_06s0004g05780	Glucosyltransferase	1948	1	1	1	1	1	1	1	1	1	1	1	1	1	1	1	1	1	1	1	19
VIT_09s0002g01080	Type II lipoxygenase	4712	1	1	1	1	1	1	1	1	1	1	1	1	1	1	1	1	1	1	1	19
VIT_00s0271g00060	(3S)-Linalool/(E)-Nerolidol synthase	2339	1	1	1	1	1	1	1	1	1	1	1	1	1	1	1	1	1	1	1	19
		count	26	27	27	25	27	24	24	23	21	25	24	24	28	27	24	21	20	20	14	451

Supplemental Table S5. The position of homologoue regions of PN40024 aroma gene cluster.

species	chr	start	end
Vzc	Vzc16	19504156	20001688
Ries	Res16	21371820	23001063
Var	Var_pri16	16022129	16474690
Vca	Vca16	21645193	25292978
Vch	Vch16	23297617	23845904
Vcs	Vcs_pri16	16900001	17400000
Ved	Ved16	19960833	21068836
Ves	Ves16	25740396	26614120
Vme	Vme16	18375703	18831730
Vmu	Vmu_pri16	14235398	14671764
Vra	Vra16	17688407	18223942
Vri	Vri16	16920783	17415245
VsyD	VsyD16	28461126	29120513
VsyE	VsyE16	22884261	23331854
VsyC	VsyC16	17237073	18313860
VsyA	VsyA16	16432592	16855927
VsyB	VsyB16	24260248	25196536
Vzc	Vzc16	19504156	20561452

Supplemental Table S6. The counts of NLR genes in each category.

Species	CC-NBS	CC-NBS -LRR	NBS	NBS-LRR	RPW8-X	TIR-NBS	TIR-NBS -LRR	TIR-X	TIR+CC	Total
**Ries**	302	127	297	73	16	88	13	125	21	1041
**Vca**	304	102	271	56	18	48	14	83	13	896
**Vch**	235	71	192	56	15	61	7	99	16	736
**Vcs**	198	61	141	39	8	48	9	55	15	559
**Ved**	303	80	246	59	9	67	10	101	27	875
**Ves**	296	83	264	57	13	51	16	79	30	859
**Vme**	228	51	282	39	6	48	3	82	10	739
**Vra**	78	28	93	16	7	29	2	40	5	293
**Vzc**	264	62	253	66	8	58	10	75	17	796
**VsyA**	227	86	178	59	14	45	8	52	11	669
**VsyB**	277	78	218	74	9	68	11	123	18	858
**VsyC**	273	82	252	74	17	64	13	96	20	871
**VsyD**	329	79	241	76	13	61	10	77	17	886
**VsyE**	296	97	268	74	11	65	7	93	25	911
**Vri**	117	58	111	29	1	88	15	66	21	485
**Var**	271	84	205	76	6	70	8	72	14	792
**Vmu**	208	91	167	52	12	59	10	82	21	681

Supplemental Table S7. The counts of NLR gene in GBPG.

population	trunk	bubbles	total	average diversity contribution
wild (n = 5)	629	1174	1803	234.8
cultivar (n = 9)	621	1458	2079	162
all (n = 17)	604	4816	5420	283.3

Supplemental Table S8. The counts of paired or clustered NLR genes.

sample	pairs num	paired genes num	paried genes proportion	clusters num	clustered genes num	clustered genes proportion
Ries	117	234	0.220338983	85	362	0.34086629
Vca	99	198	0.217821782	68	291	0.320132013
Vch	76	152	0.20212766	81	354	0.470744681
Vcs	68	136	0.236933798	48	196	0.341463415
Ved	92	184	0.203991131	75	318	0.352549889
Ves	87	174	0.195725534	72	282	0.317210349
Vme	80	160	0.213618158	53	214	0.285714286
Vra	26	52	0.174496644	7	23	0.077181208
Vzc	67	134	0.164821648	63	279	0.343173432
VsyD	94	188	0.208194906	71	306	0.338870432
VsyE	78	156	0.166666667	86	366	0.391025641
VsyC	93	186	0.208754209	83	332	0.372615039
VsyA	81	162	0.238235294	50	207	0.304411765
VsyB	83	166	0.189497717	74	333	0.380136986
Var	87	174	0.215880893	82	378	0.46898263
Vri	28	56	0.110671937	42	207	0.409090909
Vmu	53	106	0.150997151	80	367	0.522792023
